# Variations in hepatic lipid species of age-matched male mice fed a methionine-choline-deficient diet and housed in different animal facilities

**DOI:** 10.1186/s12944-019-1114-4

**Published:** 2019-09-14

**Authors:** Lisa Rein-Fischboeck, Elisabeth M. Haberl, Rebekka Pohl, Susanne Feder, Gerhard Liebisch, Sabrina Krautbauer, Christa Buechler

**Affiliations:** 1 Department of Internal Medicine I, Regensburg University Hospital, D-93042 Regensburg, Germany; 2 Institute of Clinical Chemistry and Laboratory Medicine, Regensburg University Hospital, Regensburg, Germany

**Keywords:** Ceramide, Cholesterol, Chemerin, Phosphatidylcholine

## Abstract

**Background:**

Non-alcoholic steatohepatitis (NASH) is a common disease and feeding mice a methionine-choline-deficient (MCD) diet is a frequently used model to study its pathophysiology. Genetic and environmental factors influence NASH development and liver lipid content, which was studied herein using C57BL/6 J mice bred in two different animal facilities.

**Methods:**

Age-matched male C57BL/6 J mice bred in two different animal facilities (later on referred to as WT1 and WT2) at the University Hospital of Regensburg were fed identical MCD or control chows for 2 weeks. Hepatic gene and protein expression and lipid composition were determined.

**Results:**

NASH was associated with increased hepatic triglycerides, which were actually higher in WT1 than WT2 liver in both dietary groups. Cholesterol contributes to hepatic injury but was only elevated in WT2 NASH liver. Ceramides account for insulin resistance and cell death, and ceramide species d18:1/16:0 and d18:1/18:0 were higher in the NASH liver of both groups. Saturated sphingomyelins only declined in WT1 NASH liver. Lysophosphatidylcholine concentrations were quite normal in NASH and only one of the 12 altered phosphatidylcholine species declined in NASH liver of both groups. Very few phosphatidylethanolamine, phosphatidylserine, and phosphatidylinositol species were comparably regulated in NASH liver of both animal groups. Seven of these lipid species declined and two increased in NASH. Notably, hepatic mRNA expression of proinflammatory (F4/80, CD68, IL-6, TNF and chemerin) and profibrotic genes (TGF beta and alpha SMA) was comparable in WT1 and WT2 mice.

**Conclusions:**

Mice housed and bred in different animal facilities had comparable disease severity of NASH whereas liver lipids varied among the groups. Thus, there was no specific lipid signature for NASH in the MCD model.

**Electronic supplementary material:**

The online version of this article (10.1186/s12944-019-1114-4) contains supplementary material, which is available to authorized users.

## Background

Non-alcoholic fatty liver disease (NAFLD) is a common diagnosis particularly in overweight patients. The progressive form, non-alcoholic steatohepatitis (NASH), may lead to liver cirrhosis and hepatocellular carcinoma [1, 2]. Both, hepatic steatosis and liver fibrosis, are heritable, and numerous genetic variants contribute to disease progression [3, 4]. Many of the genes identified so far have a role in lipid metabolism [3]. Liver steatosis is a characteristic feature of NAFLD and most of the accumulating lipids are triglycerides [1, 2]. Triglycerides are biologically inert lipids and protect the liver from lipotoxicity [5]. Indeed, inappropriate triglyceride synthesis in conjunction with disturbed homeostasis of further lipid classes act as harmful agents on liver cells [6].

Lipid metabolism is also influenced by the gut microbiome [7]. Intestinal bacteria modulate fat absorption, choline metabolism, bile acid and cholesterol homeostasis [7, 8]. Gut dysbiosis is related to obesity and NAFLD [9], but notably has a role in the pathophysiology of various diseases [10]. Gut bacteria composition is affected by diet, the genetic background and environmental factors like stress [11]. NAFLD risk may be also increased by infection with Helicobacter species which may alter the intestinal microbiome and gut inflammation [12].

There are several animal models to study the pathogenesis of NASH [13, 14]. Feeding rodents a methionine-choline-deficient (MCD) diet is a widely used approach [13, 14]. MCD diet induces hepatic steatosis, inflammation, and eventually liver fibrosis. Lack of methionine contributes to body weight loss, oxidative stress, inflammation and fibrosis whereas absence of choline mainly causes liver steatosis [15].

A study performed by Kim et al. compared C57BL/6 N mice originating from Korea, USA, and Japan with the MCD diet. There were no major differences of organ weights, liver steatosis and levels of alanine aminotransferase, aspartate aminotransferase, triglycerides, and cholesterol between the three mice groups [16]. Consistently, MCD diet induced hepatic triglyceride accumulation in different studies. However, NASH-associated changes of further lipid classes were less reproducible [17–19].

In NASH, hepatocytes displaying cholesterol crystals activated circumjacent Kupffer cells and thus contributed to disease progression [20]. In mice fed a MCD chow, liver cholesterol was increased in some but not all models [18, 19]. The ratio of hepatic phosphatidylcholine (PC) to phosphatidylethanolamine (PE) is important for membrane integrity and liver health, and was decreased in NASH [21]. Not all studies could identify reduced phosphatidylcholine in the liver of MCD diet fed rodents [17–19, 22].

Particular ceramide species are cytotoxic and contribute to liver cell death [23]. After all, hepatic ceramide levels were unchanged or induced in the liver of MCD diet fed mice [18, 19, 22]. High, low and normal sphingomyelin levels were detected in NASH liver [18, 19, 22]. In summary, results regarding changes of hepatic lipids in the different MCD models were inconsistent [17, 18, 22, 24].

Differences in gender, age, composition of the diets and feeding period most likely contributed to these variations [17, 18, 22, 25]. Breeding of mice in different facilities caused genetic divergences, and this may also modulate hepatic lipid composition and NASH progression [3, 26]. Rodents in animal facilities with unrestricted access had a higher bacterial diversity [27]. Gut microbiome composition changed when different rodents were housed in an animal facility [27]. Thus, environmental factors affect the gut microbiome, which may also influence NASH pathogenesis and hepatic lipid composition [7, 9].

Here we hypothesized that mouse substrains housed in separate animal facilities differentially respond to MCD diet. Therefore, age-matched male mice were fed identical control chows or MCD diets for 2 weeks. Hepatic expression of NASH-specific genes and liver lipid composition were determined.

## Materials and methods

### Animal handling

The male C57BL/6 J mice were originally from the Jackson Laboratory (Bar Harbor, USA). Mice of the parent generation which were bred for at least 20 generations in two different conventional animal units at the University Hospital of Regensburg are referred to as wild type (WT) 1 and WT2 animals. Fifteen mice (14 weeks old - 8 WT1 and 7 WT2) were fed the MCD diet (E15653–94, Ssniff, Soest, Germany) and eleven mice (14 weeks old - 6 WT1 and 5 WT2 animals) the respective control chow (E15654–04, Ssniff, Soest, Germany) for 2 weeks. Experiments involving WT1 mice were performed in October 2014 and studies with WT2 mice in August 2016. Composition of the diets was identical over time (personal communication with Ssniff). Body weights were measured shortly before the experiment, 7 and 14 days later. The animals were fasted for 12 h and were all killed between 8 and 10 am. Livers were excised and stored in fluid nitrogen immediately. To avoid refreezing of the samples aliquots of liver tissues were stored at − 80 °C until used for the experiments. Storage of tissues at this temperature for 7 months did not cause degradation of lipids [28].

Animal and laboratory experiments were done by the same researchers. Health monitoring in the animal facilities was performed in accordance with the recommendations of the Federation of European Laboratory Animal Science Associations (FELASA) [29]. Mouse hepatitis virus and *Helicobacter spp*. were detected in the facility where WT2 mice were housed but were never identified in the WT1 facility. Both facilities were barrier-free. Room temperature, light dark cycle, humidity, cages, bedding, and standard chows were identical. Further, number of mouse in a cage was similar. Different animal keepers were employed in the two facilities. While in facility 1 only rats and mice were housed there were also rabbits in facility 2. Mouse weighing can be done in the room where the cages were located in facility 1 whereas this was done in a separate room in facility 2.

### Quantification of lipids

Lipid quantification was done by direct flow injection electrospray ionization tandem mass spectrometry (ESI–MS/MS) in positive ion mode as described in detail elsewhere [19, 30–32].

Quantification of all lipid classes was based on standard addition calibration, which includes various species to address response differences [32]. Total concentrations of the lipid classes were obtained by addition of the values of the single species. Lipid analysis of all livers was done in parallel. Lipid species are given in compliance with the published proposal for shorthand notation of lipid structures analyzed by mass spectrometry [33]. Glycerophospholipid notation assumes the presence of even numbered carbon chains. Sphingomyelin species annotation implicates that a sphingoid base d18:1 is present. Saturated species have no double bond in the amide linked fatty acid chain and the monounsaturated species have one double bond. Liver lipids are all given as nmol/mg wet weight. Lipid species where values of all animals were below 0.01 nmol/mg wet weight were regarded as doubtful and were not used. Data of WT1 mice have been partly published [19].

Triglycerides were measured with the colorimetric triglyceride quantification kit from BioVision (Heidelberg, Germany).

### Oil red O staining

For the detection of neutral lipids, 5 μm-thick cryosections were fixed with formalin and stained with 0.3 g / 100 ml Oil Red O (Sigma, Taufkirchen, Germany) solution for 15 min at room temperature and then counterstained with hematoxylin for 1 min.

### SDS-PAGE and immunoblotting

SDS-PAGE and immunoblotting were performed as described [30, 34]. Antibody for GAPDH was from New England Biolabs GmbH (Frankfurt am Main, Germany). Chemerin antibody was from R&D Systems (Wiesbaden, Germany).

### Semiquantitative real-time PCR

Semiquantitative real-time PCR (LightCycler® FastStart DNA *Master* SYBR Green I kit from Roche, Mannheim, Germany) was performed as described [34]. Serial dilutions of cDNA (obtained from murine, hepatic RNA) were amplified in parallel to the respective samples to obtain a standard curve. Calculation of mRNA expression was done relative to the values of the standards. This approach is appropriate to correct for different PCR efficiencies, which have to be considered when analyzing different genes. Further, by using identical standards, PCRs performed at different times can be compared. Analysis of gene expression of mice fed the MCD diet and the respective controls was done at the same time. Primers to amplify F4/80, TGF beta, alpha SMA, CD68 and 18S rRNA were used as published recently [19]. TNF was analyzed as described [35]. Primers 5’ttc cat cca gtt gcc ttc tt 3′ and 5’ttc tgc aag tgc atc atc gt 3’were used for IL-6 analysis. Data of the WT1 animals have been published earlier [19].

### Analysis of malondialdehyde

Malondialdehyde (Lipid Peroxidation (MDA) Assay Kit) was measured by a colorimetric assay from Abcam (Cambridge, UK).

### Statistical analysis

Data are shown as boxplots (median value, the lower and upper quartiles and the range of the values). Statistical analysis was done by Kruskal-Wallis test (SPSS Statistics 25.0 program, IBM, Leibniz Rechenzentrum, München, Germany). A value of *p* < 0.05 was regarded as significant.

## Results

### Body weight change, fat pad weight and liver weight

**Fig. 1:**
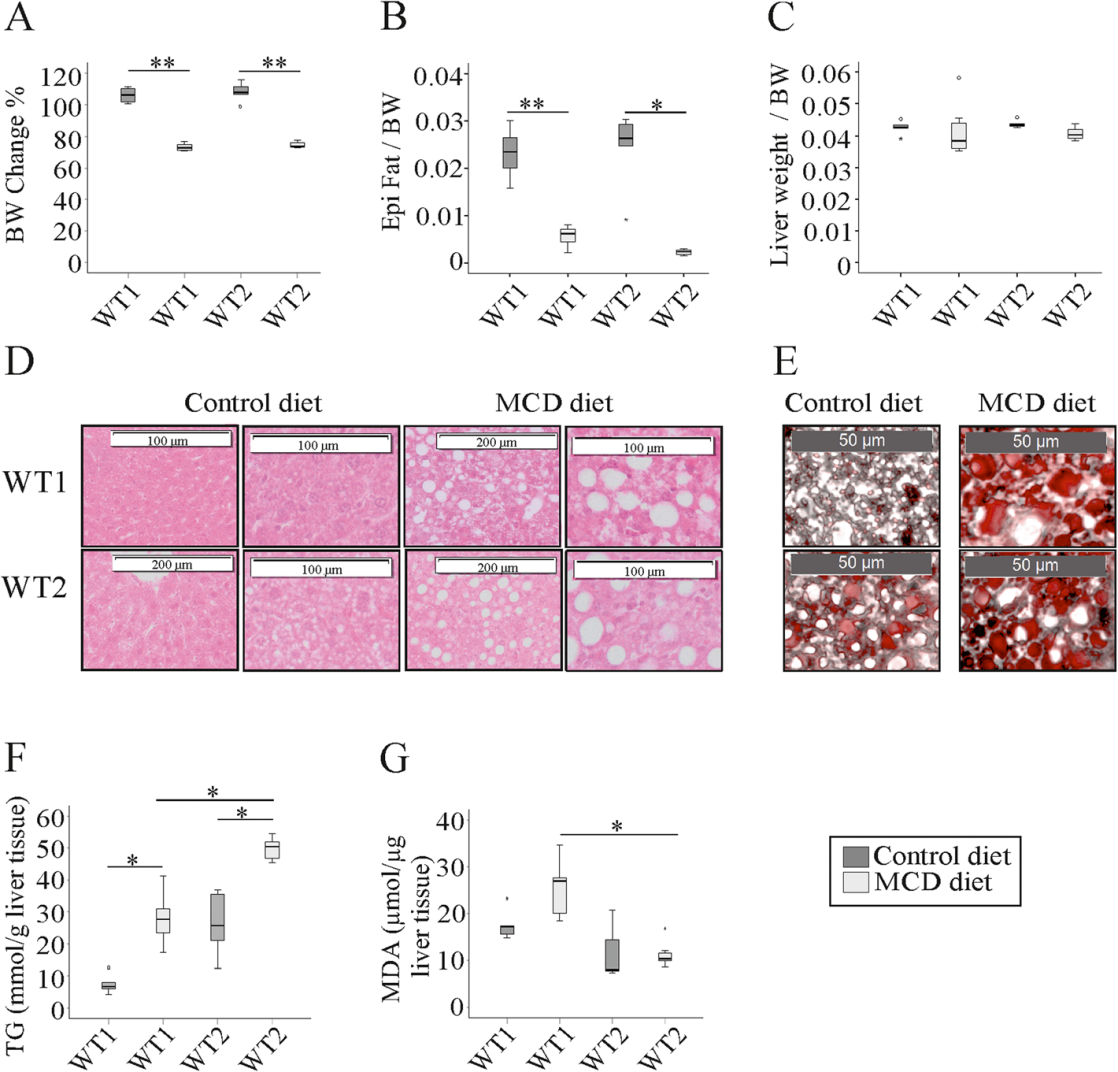
Body weight change, fat pad weight, liver weight, and hepatic triglyceride (TG) and malondialdehyde (MDA) levels in mice fed a methionine-choline-deficient (MCD) or control diet and housed in two different animal facilities (WT1, WT2).**a** Body weight (BW) at the end of the study relative to the initial weight in %. **b** Epididymal (Epi) fat pad weight normalized to BW. **c** Liver weight normalized to BW. **d** H&E stained liver of WT mice fed a control diet. Small lipid droplets appear in the liver of WT2 mice and are visible at higher magnification (second row). Large lipid droplets in the liver of MCD diet fed mice (third and fourth row). **e** Oil Red O stained liver. **f** Hepatic TG concentrations. **g** Hepatic MDA levels. Data of 5 to 8 animals per group are shown. * *p* < 0.05, ** *p* < 0.01

Age-matched male C57BL/6 J mice housed in two different animal facilities at the University Hospital of Regensburg were fed the identical MCD diet or control chow for 2 weeks. In accordance with previous studies all of the mice fed the MCD diet lost body weight [14] without any differences between the two groups (Fig. 1a). Body weight change during these 2 weeks did not vary between mice kept in the different animal facilities. This suggests that food intake, although not measured, was similar. Accordingly, subcutaneous, epididymal and perirenal white adipose tissue weights were similarly reduced in both groups (Fig. 1b and data not shown). Liver to body weight ratios were comparable in all of the mice (Fig. 1c). In the liver of WT2 mice fed the control chow small lipid droplets were visible which were not detected in WT1 mouse liver. Large lipid droplets appeared in the liver of WT1 and WT2 mice fed the MCD diet (Fig. 1d, e). Liver triglyceride concentrations of both groups increased in NASH. Hepatic triglycerides tended to be higher in WT2 than WT1 mice fed the control chow and were significantly increased in WT2 compared to WT1 NASH liver (Fig. 1f). Malondialdehyde (MDA) levels as a surrogate marker of lipid peroxidation were higher in WT1 NASH liver when compared to WT2 NASH liver (Fig. 1g).

### Hepatic gene and protein expression

**Fig. 2:**
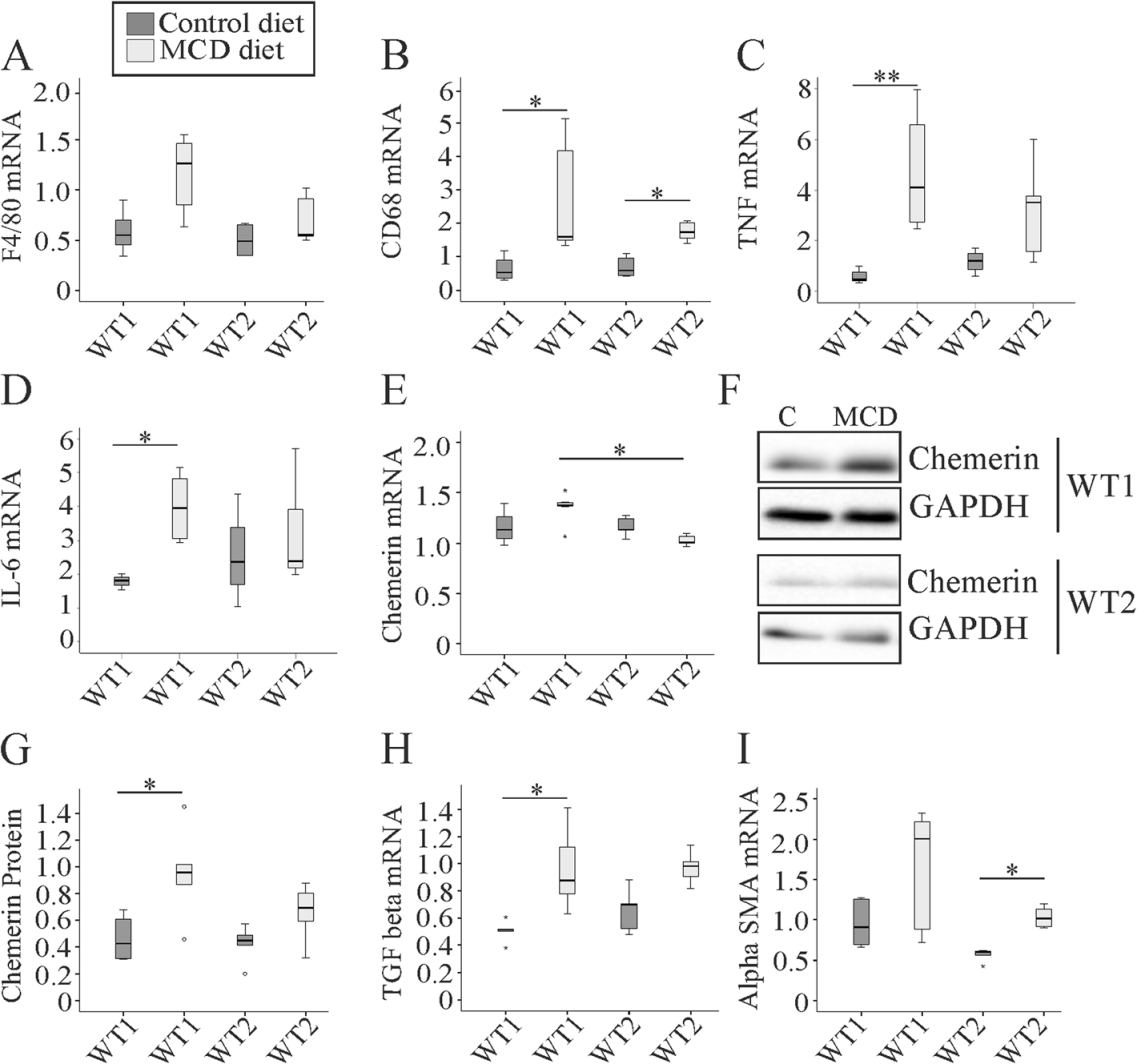
Hepatic expression of inflammatory and profibrotic genes in mice fed a methionine-choline-deficient (MCD) or control diet and housed in two different animal facilities (WT1, WT2).**a** Hepatic F4/80 mRNA. **b** Hepatic CD68 mRNA. **c** Hepatic TNF mRNA. **d** Hepatic IL-6 mRNA. **e** Hepatic chemerin mRNA. **f** Hepatic chemerin protein. **g** Quantification of hepatic chemerin protein. **h** Hepatic TGF beta mRNA. **i** Hepatic alpha SMA mRNA. Data of 5 to 8 animals per group are shown. * *p* < 0.05, ** *p* < 0.01

F4/80 and CD68 are genes expressed by macrophages and were induced in NASH liver [36]. CD68 mRNA expression was upregulated in NASH liver of both animal groups whereas F4/80 was not significantly changed (Fig. 2a, b). The mRNA levels of tumor necrosis factor (TNF) and interleukin-6 (IL-6) only increased significantly in WT1 NASH liver (Fig. 2c, d). The mRNA expression of chemerin, a chemoattractant for immune cells [37], was not changed in NASH liver (Fig. 1e). Hepatic chemerin protein only increased in the liver of WT1 MCD diet fed mice (Fig. 2f, g). Regarding expression of profibrotic genes, the mRNA levels of alpha-smooth muscle actin (SMA) and transforming growth factor (TGF) beta showed similar trends in both groups (Fig. 2h, i). Large variability in gene / protein expression most likely accounts for the lack of significance in some of the experiments.

### Hepatic cholesterol species

**Fig. 3:**
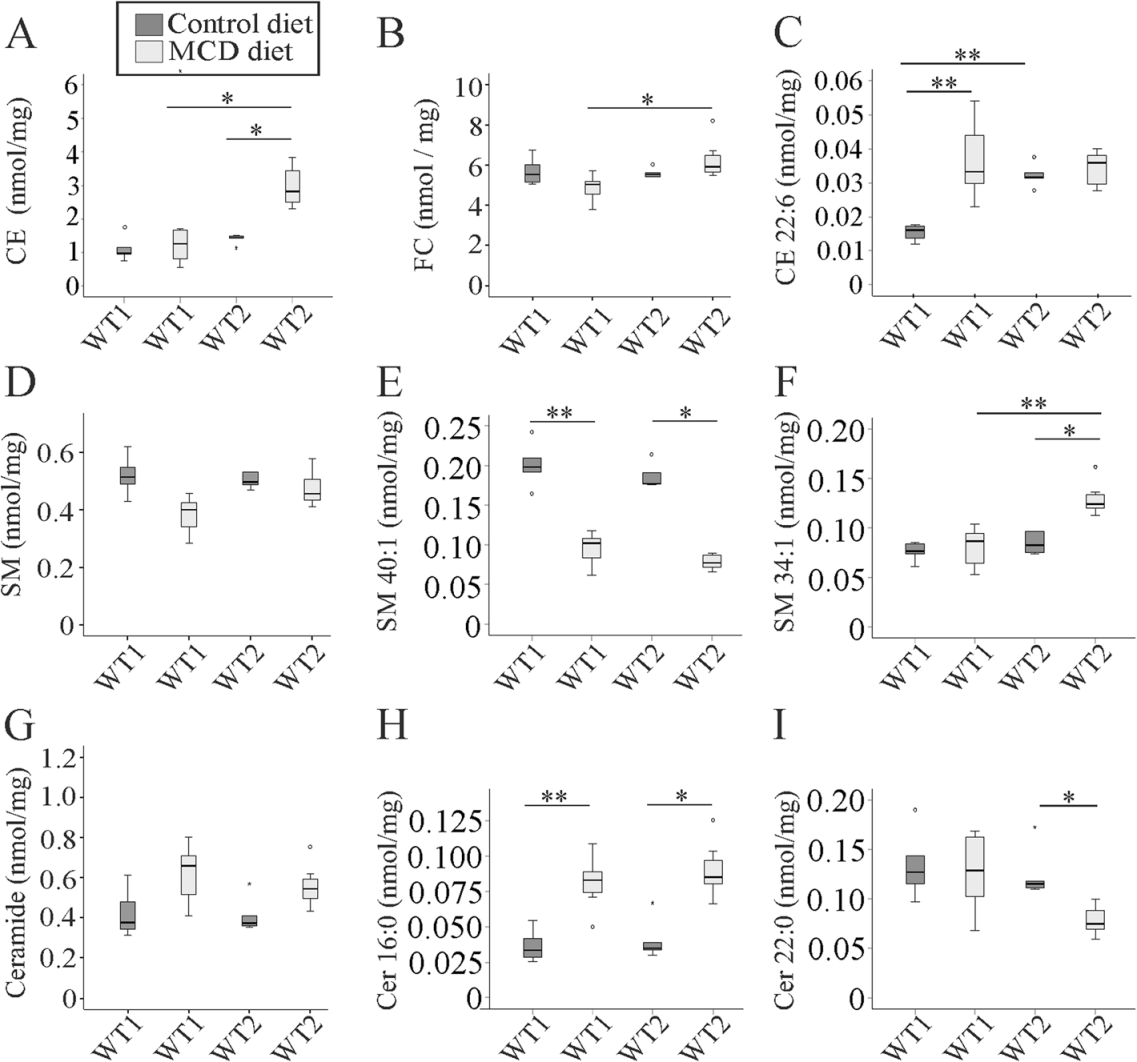
Cholesteryl ester (CE), free cholesterol (FC), sphingomyelin (SM) and ceramide (Cer) in the liver of mice fed a methionine-choline-deficient (MCD) or control diet and housed in two different animal facilities (WT1, WT2). Concentrations are given as nmol/mg wet weight. **a** CE **b** FC **c** CE 22:6 **d** Total SM **e** SM 40:1 **f** SM 34:1 **g** Total Cer **h** Cer d18:1/16:0 and (**i**) Cer d18:1/22:0. Data of 5 to 8 animals per group are shown. * *p* < 0.05, ** *p* < 0.01

Excess hepatic cholesterol contributes to cell death, inflammation and fibrosis in NASH [38]. Indeed, total levels of cholesteryl ester (CE) increased in the NASH liver of WT2 mice. This effect was not identified in WT1 mice. As a result, CE was higher in WT2 than WT1 NASH liver (Fig. 3a). Free cholesterol (FC) was unchanged in NASH liver but was higher in WT2 than WT1 NASH liver (Fig. 3b and Additional file 1: Table S1C).

Calculations of individual CE species revealed that simply CE 22:6 increased in WT1 NASH liver (Fig. 3c and Additional file 1: Table S1B). The CE species CE 14:0, 16:0, 18:3, 18:2, 18:1 and 18:0 were just induced in WT2 NASH liver (Additional file 1: Table S1A, B). In fact, the CE species CE 14:0, 16:1, 16:0, 18:3, 18:2 and 18:1 were high in WT2 NASH liver compared to WT1 NASH liver (Additional file 1: Table S1A). Interestingly, CE 18:3, 18:2 (Additional file 1: Table S1A) and CE 22:6 (Fig. 3c, Additional file 1: Table S1B) levels were more abundant in the liver of WT2 than WT1 controls.

### Hepatic sphingolipids

Sphingolipids regulate various signaling pathways in NASH [39, 40]. Analysis of sphingomyelin (SM) levels in MCD-NASH livers showed divergent results [18, 19, 22]. Current analysis revealed that SM levels were not significantly changed in NASH liver of both groups (Fig. 3d). Saturated SM species were low in WT1 NASH liver compared to the respective control animals (Additional file 1: Table S2B).

In WT1 and WT2 NASH liver SM 40:2 (Additional file 1: Table S2A) and SM 40:1 (Fig. 3e and Additional file 1: Table S2A) were low whereas SM 36:1 (Additional file 1: Table S2A) was even high when compared to the respective control mice. In WT2 mouse liver SM 34:1 increased in NASH (Fig. 3f and Additional file 1: Table S2A). Comparison of SM species in WT1 and WT2 NASH liver revealed lower SM 34:1 (Fig. 3f and Additional file 1: Table S2A), 36:1 and 42:2 concentrations in the first group (Additional file 1: Table S2A). SM36:1 was further low in WT1 mice when compared to the respective WT2 mice fed the control chow (Additional file 1: Table S2A).

Degradation of SM can produce ceramide (Cer) which was found increased in NASH liver [17]. Unexpectedly, saturated (Additional file 1: Table S3B) and total Cer levels (Fig. 3g) were not induced in NASH liver. Cer d18:1/16:0 (Fig. 3h and Additional file 1: Table S3A) and Cer d18:1/18:0 (Additional file 1: Table S3A) increased in WT1 and WT2 NASH liver. In the WT2 mice Cer d18:1/23:0 and d18:1/24:1 were also higher (Additional file 1: Table S3A) whereas Cer d18:1/22:0 declined (Fig. 3i and Additional file 1: Table S3A). Comparison of the lipidome of WT1 and WT2 NASH liver revealed higher Cer d18:1/18:0 in the latter (Additional file 1: Table S3A). Of note, ceramide species were comparable in WT1 and WT2 controls (Fig. 3h, i and Additional file 1: Table S3A).

SM 40:1 is the most abundant SM species in the liver and was reduced in NASH (Fig. 3e and Additional file 1: Table S2A). The corresponding sphingomyelinase derived Cer species (Cer d18:1/22:0) was, however, not significantly changed in WT1 and even declined in WT2 NASH liver (Fig. 3i and Additional file 1: Table S3A). Cer d18:1/16:0 and the corresponding species SM 34:1 were both increased in NASH liver of WT2 mice (Fig. 3f, h and Additional file 1: Table S2A, S3A). This clearly illustrates that hepatic sphingomyelinase activity was not enhanced in the murine MCD models studied herein.

### Hepatic lysophosphatidylcholine and phosphatidylcholine

**Fig. 4:**
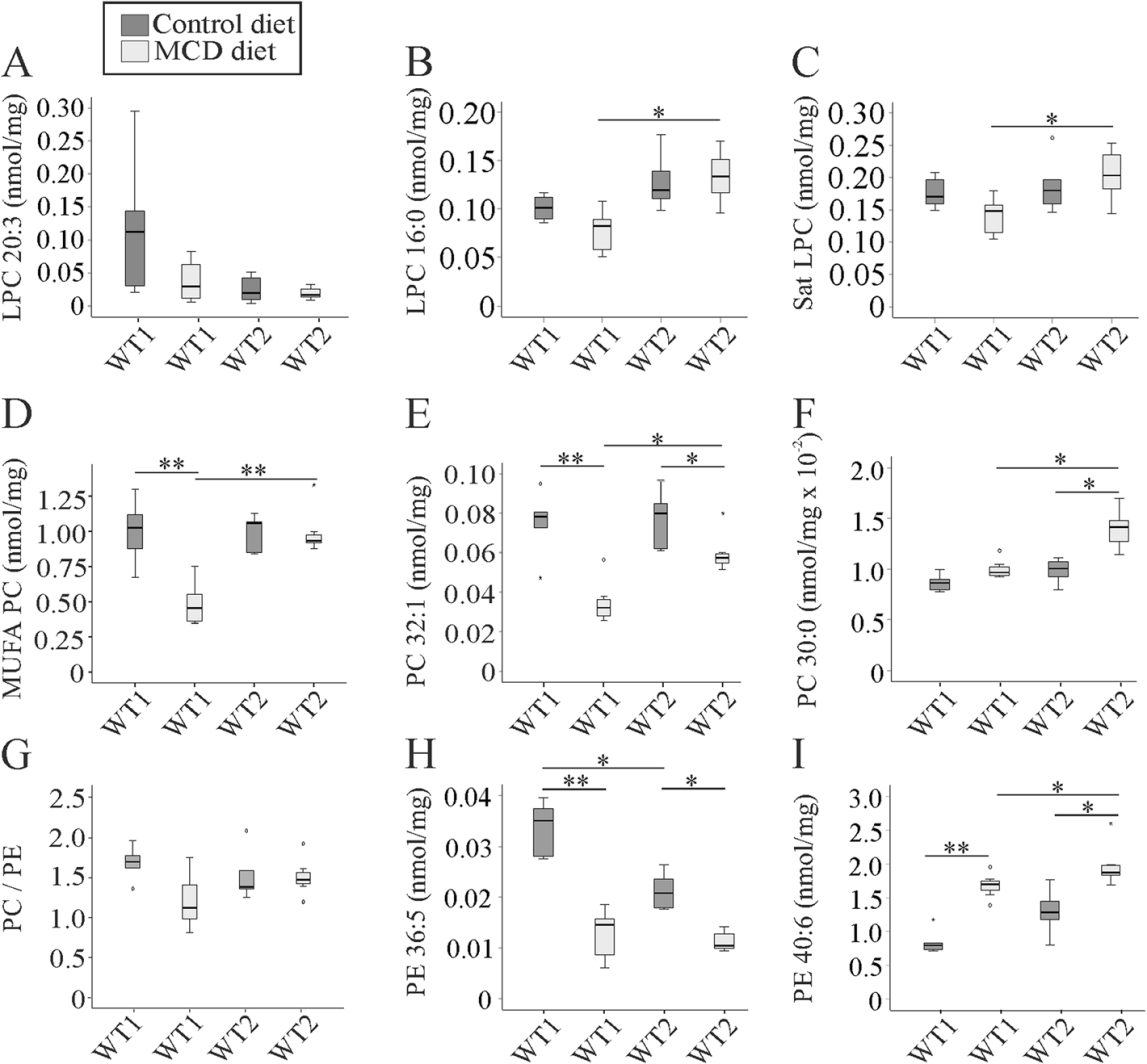
Lysophosphatidylcholine (LPC), phosphatidylcholine (PC) and phosphatidylethanolamine (PE) in the liver of mice fed a methionine-choline-deficient (MCD) or control diet and housed in two different animal facilities (WT1, WT2). Concentrations are given as nmol/mg wet weight.**a** LPC 20:3 **b** LPC 16:0 **c** saturated (Sat) LPC **d** monounsaturated (MUFA) PC **e** PC 32:1 **f** PC 30:0 **g** PC to PE ratio **h** PE 36:5 and **i** PE 40:6. Data of 5 to 8 animals per group are shown. * *p* < 0.05, ** *p* < 0.01

Lysophosphatidylcholine (LPC) was found to contribute to lipoapoptosis in palmitate incubated hepatocytes [41]. Surprisingly, not a single LPC species was significantly changed in NASH liver (Fig. 4a - c and Additional file 1: Table S4A, B). All of the LPC species measured were similarly abundant in livers of WT1 mice and WT2 mice on a standard chow (Fig. 4a - c and Additional file 1: Table S4A, B). Compared to WT1 MCD diet fed mice LPC 16:0 (Fig. 4b and Additional file 1: Table S4A) and LPC 18:1 (Additional file 1: Table S4A), and thus saturated and monounsaturated LPC, were higher in WT2 NASH liver (Fig. 4b, c and Additional file 1: Table S4B, C).

Phosphatidylcholine (PC) has a central role in the export of liver lipids to the blood [42]. Whereas monounsaturated PC was low in the NASH liver of WT1 animals compared to the controls, this was not the case in WT2 mice (Fig. 4d and Additional file 1: Table S5D). Species which were lower in WT1 NASH liver than controls (PC 32:1, 34:1, 36:1, 38:3, 38:2, 38:1, 40:4) were - with the exception of PC 32:1 (Fig. 4e and Additional file 1: Table S5A) - normal in the WT2 animals. Here, PC 30:0 (Fig. 4f and Additional file 1: Table S5A), PC 32:2, 36:3, 36:2 and 40:5 were actually induced (Additional file 1: Table S5A, B, D). Thus, PC 30:0 (Fig. 4f and Additional file 1: Table S5A), PC 32:2 (Additional file 1: Table S5A), PC 32:1 (Fig. 4e and Additional file 1: Table S5A), PC 34:1, 36:3, 36:2, 36:1 and 40:5 were high in WT2 NASH liver in comparison to WT1 NASH liver (Additional file 1: Table S5A - D). PC species 36:5, 36:4, 36:2, 38:3 and 40:5 were diminished in the liver of WT2 mice relative to WT1 animals kept on the control chow (Additional file 1: Table S5B - D).

Therefore, PC 32:1 was the only species concordantly changed in the NASH liver of WT1 and WT2 mice (Fig. 4e and Additional file 1: Table S5A).

### Hepatic phosphatidylethanolamine, phosphatidylserine and phosphatidylinositol

Decreased PC to phosphatidylethanolamine (PE) ratio is associated with impaired membrane integrity and liver injury [21]. This ratio was not changed upon NASH development in both animal groups (Fig. 4g).

Total PE concentrations were not changed in NASH liver, and furthermore, were comparable between the mice housed in different facilities (data not shown). Various individual PE species (PE 36:5 (Fig. 4h and Additional file 1: Table S6A), PE 36:4, 38:5, 38:4, 38:3, 38:2, 40:4, 40:3, 42:6 (Additional file 1: Table S6A - C) were nevertheless reduced in the NASH liver of WT1 animals. Anyhow, PE 40:6 was induced in WT1 and WT2 NASH liver (Fig. 4i and Additional file 1: Table S6C). In the WT2 NASH liver PE 36:1, 40:7, 40:6 and 40:5 were also higher whereas PE 34:2 and 36:5 were low when compared to the WT2 controls (Additional file 1: Table S6A - C). In contrast to WT1 NASH liver, PE 36:3, 36:2, 36:1 (Additional file 1: Table S6A - B), PE 40:6 (Fig. 4i and Additional file 1: Table S6C) and PE 40:5 (Additional file 1: Table S6C) were higher in WT2 mice.

PE 36:5 (Fig. 4h and Additional file 1: Table S6A), PE 38:5. 38:3, 38:2, 40:4 and 42:6 were lower in the liver of WT2 compared to WT1 mice fed the control chow (Additional file 1: Table S6A - D).

Of the 20 analyzed PE species solely PE 36:5 and 40:6 were comparable changed in NASH liver of both cohorts (Fig. 4h, i).

**Fig. 5:**
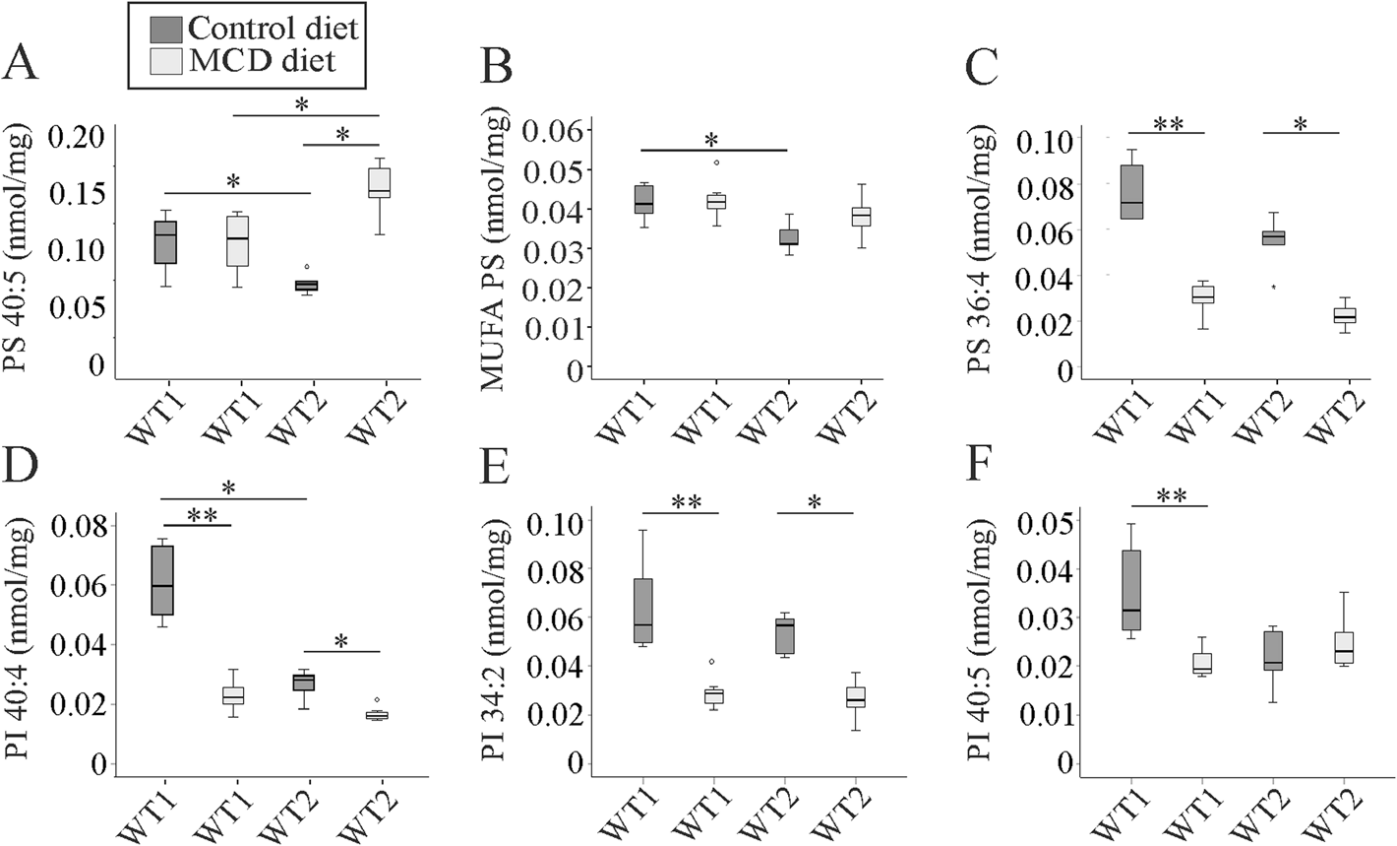
Phosphatidylserine (PS) and phosphatidylinositol (PI) in the liver of mice fed a methionine-choline-deficient (MCD) or control diet and housed in two different animal facilities (WT1, WT2). Concentrations are given as nmol/mg wet weight. **a** PS 40:5 **b** Monounsaturated (MUFA) PS **c** PS 36:4 **d** PI 40:4 **e** PI 34:2 and **f** PI 40:5. Data of 5 to 8 animals per group are shown. * *p* < 0.05, ** *p* < 0.01

Phosphatidylserine (PS) levels differed markedly in the liver of mice fed the control chow. PS 36:2, 38:5, 38:4, 38:3 (Additional file 1: Table S7A, B), PS 40:5 (Fig. 5a and Additional file 1: Table S7B) and MUFA PS (Fig. 5b and Additional file 1: Table S7B) were higher in WT1 mice. In NASH liver PS 40:5 was the only species which differed between the two groups, and was higher in WT2 compared to WT1 mice (Fig. 5a and Additional file 1: Table S7B).

While most PS species (PS 36:4, 38:5, 38:4, 38:3) declined in WT1 NASH liver compared to the respective control animals, PS 40:6 was induced (Additional file 1: Table S7A, B). In WT2 animals PS 36:4 (Fig. 5c and Additional file 1: Table S7A) and PS 38:4 (Additional file 1: Table S7A) were reduced and PS 36:2, 40:6 (Additional file 1: Table S7A, B) and PS 40:5 (Fig. 5a and Additional file 1: Table S7B) were increased. Hence, three of the 11 measured PS species (PS 36:4 (Fig. 5c and Additional file 1: Table S7A), 38:4 and 40:6 (Additional file 1: Table S7A, B) were concordantly changed in NASH liver of both animal groups.

Several phosphatidylinositol species namely PI 38:5, 38:3 (Additional file 1: Table S8B) and PI 40:4 (Fig. 5d and Additional file 1: Table S8B) were low in WT2 compared to WT1 liver in the control group. In the MCD diet fed animals there was no difference in any of the hepatic PI species (Fig. 4d - f and Additional file 1: Table S8A, B). In NASH liver PI 34:2 (Fig. 5e), PI 36:4 (Additional file 1: Table S8A), PI 38:3 (Additional file 1: Table S8B) and PI 40:4 (Fig. 5d and Additional file 1: Table S8B) decreased in both animal groups. PI 38:5 (Additional file 1: Table S8B) and PI 40:5 (Fig. 5f) only declined in WT1 mice in NASH. Therefore, four (PI 34:2, PI 36:4, PI 38:3, PI 40:4) of the 12 analyzed PI species declined in the NASH liver of all of the animals.

## Discussion

Lipidomics uses techniques such as mass spectrometry and chromatography to quantify different lipid species and was applied to analyze lipid composition of NASH liver [43–45]. The present study illustrated that mice bred and housed in two different animal facilities showed marked differences in their hepatic lipidome when fed a NASH inducing diet. Very few lipid species were similarly altered in NASH liver of both groups and may be causally linked to disease pathogenesis. Of note, NASH disease severity did not differ between the groups.

Mice fed the MCD diet had increased triglycerides in the liver [22, 46, 47] and this was also found in the present study. WT2 mice had about two-fold higher triglyceride levels than WT1 animals irrespective of the diet. Lipid peroxidation as a marker of oxidative stress was markedly lower in the NASH liver of the WT2 mice. Further, the expression of inflammatory genes tended to be less prominent in WT2 NASH liver. Attempts to block hepatic triglyceride storage caused increased oxidative stress, inflammation and liver injury [5] showing that appropriate triglyceride synthesis is a protective mechanism in the steatotic liver. Lower triglyceride levels in WT1 mice may thus favor inflammation and fibrosis in NASH liver.

Upregulated and unchanged IL-6 mRNA expression in MCD-NASH liver were identified in the different mouse groups analyzed herein and was also reported in separate studies by others [46, 47]. Chemerin was found increased in NASH liver of MCD diet fed rodents [48] and was also higher in one of the present models. The analyzed genes and chemerin protein were, however, similarly expressed in WT1 and WT2 NASH liver suggesting that disease severity did not markedly differ between the two groups.

The two so far published comprehensive human studies analyzing the liver lipidome described a decline of hepatic PC levels in NASH liver [43–45]. Total PC was not reduced in the animals studied herein, and PC 32:1 was the only PC species concordantly reduced in the liver of WT1 and WT2 mice upon MCD diet. Choline deficient chows could not deplete hepatic PC levels, and synthesis by the CDP-choline pathway and PE methylation was normal [49, 50]. NASH development was not advanced by low hepatic PC levels in rodents [51]. The ratio of PC to PE is an established marker of membrane integrity [21] but was normal in the NASH liver of the mice studied herein.

In WT1 NASH mice various PC and PE species were reduced. Indeed, only PE 36:5 was diminished whereas PE 40:6 was induced in the NASH liver of both animal groups. Another study by Najt et al. could not identify higher PE 40:6 in MCD-NASH liver whereas PE 36:5 was not measured [18]. Therefore, these two PE species can not be considered as to be causal for NASH pathogenesis. Likewise, lipidomic analysis of human NASH livers revealed discordant results. One study showed a decrease of the phospholipid classes PE, PI and PS in NASH liver whereas levels were quite normal in the NASH patients enrolled in a second analysis [43, 45]. Total hepatic PS levels were also unaffected in the MCD diet fed mice studied herein. A total number of 11 PS species was analyzed and three of them were concordantly changed in NASH liver of both animal groups (PS 36:4, 38:4, 40:6). PS 40:6 was about two-fold higher in the NASH liver. Najt et al. even described a more than 20-fold increase of this lipid species. PS 36:4 and 38:4 were not changed in their experimental model [18]. This principally argues against a causal connection between these individual phospholipid species and NASH.

Phosphatidylinositol species mostly declined in NASH liver and four of the 12 measured molecules were reduced in WT1 and WT2 NASH liver. Three of the five analyzed PI species were also low in the NASH liver analyzed in a separate study [18]. Diminished PI levels were further reported in human NASH [43] and future studies have to clarify whether the decline of these lipids has a causal role in NAFLD progression.

LPC species were not regulated in MCD-NASH liver. A separate analysis nevertheless identified strongly elevated LPC in the liver of MCD diet and high fat diet fed mice [52]. This suggests that hepatic LPC levels were modified by diet, genetics and / or housing conditions [53] but were not mandatory for NASH pathogenesis.

Total SM concentration was depleted in human NASH liver in one study while a second analysis described normal levels [43, 45]. Total SM concentrations were not changed in the experimental model studied herein. Three SM species were concordantly altered in NASH liver of both groups and SM 40:2 was similarly changed in the MCD diet fed mice in a separate study [18]. Ceramide content was found higher in human NASH liver whereas a separate study described unchanged or lower levels [39, 43].

Specific hepatic sphingolipid species were associated with insulin resistance in patients with NASH [39, 54]. Cer d18:1/16:0 produced by ceramide synthase 6 is a key metabolite in obesity associated insulin resistance [55]. This lipid was induced in NASH liver of both mouse groups studied. However, MCD diet associated weight loss improves insulin sensitivity [56] demonstrating that the nearly 2.5 fold hepatic increase of this ceramide species may not suffice to cause insulin resistance.

A common variant in patatin-like phospholipase domain containing protein 3 (I148M) was associated with hepatic steatosis whereas insulin response was quite normal [57]. Though concentrations of hepatic ceramides and saturated fatty acids, which were supposed to contribute to liver disease, were comparable to the respective controls, these patients developed NASH [54, 57]. This principally challenges the causative role of individual hepatic lipids like distinct ceramide species herein.

Cholesterol and especially FC is supposed to contribute to hepatocyte death and NASH [38]. FC was normal in the liver of the MCD diet fed mice and CE levels were only higher in WT2 NASH liver. Individual CE species were changed in WT1 and WT2 liver whereas a common effect in NASH was not been identified. FC contributes to lipotoxicity, inflammation and fibrogenesis and was found increased in human NASH [38, 45] and in WT2 NASH liver. Comparable hepatic expression of inflammatory and profibrotic genes in WT1 and WT2 NASH liver excluded differences in hepatic injury. Cholesterol may thus have a minor if any role in MCD diet induced hepatic injury. Human analysis described a gradual increase of C16 and C18 CE species from normal to steatotic to NASH liver [43]. Though cholesterol may contribute to NASH pathogenesis in a subgroup of patients its role in murine NASH seems to be different. In mice the majority of systemic cholesterol is carried in high density lipoprotein while in humans it is mostly associated with low density lipoprotein [58] indicating major differences in human and rodent cholesterol metabolism.

Present study showed that most of the lipids were not concordantly changed in the two groups of mice though both developed NASH. CD68 as a marker of inflammation [59] and TGFbeta indicating liver fibrosis [60] were comparably induced in the liver of the MCD diet fed mice. There was no difference in hepatic chemerin protein, hepatic F4/80, TNF, IL-6 or alpha SMA mRNA in the NASH liver of WT1 and WT2 mice. Further, body weight and fat mass loss which contribute to liver steatosis and hepatic injury [61] were similar. In NASH liver of WT1 and WT2 mice about 50% of CE and SM species, nearly 30% of PC and PE species, 20% of LPC species and 10% of Cer and PS species differed between the two groups whereas levels of PI species were similar. Thus, PI composition may indeed contribute to NASH pathogenesis, a suggestion which has to be examined in future studies.

Current results using an experimental model and findings in human NASH liver argue against a specific NASH lipid signature [44]. Rather genetic and environmental factors seem to greatly affect the hepatic lipidome. One future experiment is to analyze the hepatic lipidome in both experimental diet groups of genetically identical mice housed in different facilities.

Breeding of animals in separate facilities generates substrains with 0.7–1.2 coding polymorphism for a year of separation [26] which may contribute to variations between the groups. Environmental factors differ in the two facilities and may affect the lipidome.

One obvious difference between the two experimental groups was infection with mouse hepatitis virus and Helicobacter spp. in one of the facilities. The virus causes an acute and self-limiting infection without clinical symptoms in adult mice [62]. Infected animals may have an altered immunological response which indeed can affect NASH pathogenesis [62]. Helicobacter spp. infections are subclinical at least in the relatively young mice studied [63]. Expression of inflammatory genes was, however, similar in both groups kept on the control and MCD diet. Histochemistry could not demonstrate abundant infiltration of immune cells into the liver. The mice used in the current study were either not infected or exhibited very mild disease. Whether infection with these pathogens has any effect on the liver lipidome is still unknown.

Liver samples of WT1 mice were stored for about 2 years longer than tissues obtained from WT2 mice. Storage of tissues at − 80 °C for 7 months did not cause any harm to cellular lipids [28]. Patients’ samples used for lipidomic analysis were collected within one to 2 years and an effect of storage time on data quality was not reported [64].

Regarding that mice fed the identical MCD diets display high lipidomic variability it is not surprising that very few LPC species were concordantly changed when serum levels of mice fed different high fat diets in three distinct animal facilities were compared [65]. Variations of lipids were also identified in the liver of mice fed the control diet in the current study. Nearly 45% of PS species varied in the two groups, and about 25 to 33% of the analyzed CE, PC, PE and PI species were also significantly altered. This shows a high diversity of the “normal” hepatic lipidome of mice fed the identical chows which may become more variable when feeding different diets [66, 67].

Analysis of the fecal microbiome revealed that various factors like vendor, chow, access to the barrier and even housing of different rodents in the facility significantly affected its composition [27]. Microbiota was not analyzed in this study but has to be considered in future research as a cause of variations in hepatic lipid composition.

## Conclusions

Present study showed that hepatic lipid composition markedly differed in a commonly used NASH model when animals were breed and housed in different facilities. Current finding argues against a defined NASH lipid signature and postulates that several different NASH lipidomes exist.

## Additional file

Additional file 1: Tables. Lipids in the liver of WT1 and WT2 mice fed a control diet (WT1, WT2) or MCD diet (WT1 MCD, WT2 MCD). Concentrations are given as nmol/mg wet weight. The *p*-values C-MCD WT1 and C-MCD WT2 apply to the comparisons of normal to NASH liver in either WT1 or WT2 groups. C is the comparison of lipid species in the livers of WT1 and WT2 mice fed the control chow and MCD is the comparison of lipid species in the livers of WT1 and WT2 mice fed the MCD diet. * *p* < 0.05, ** *p* < 0.01. Monounsaturated, MUFA; polyunsaturated, PUFA; Sat; Saturated, **Table S1**a-c. Cholesteryl ester (CE) in the liver **Table S2**a, b Sphingomyelin (SM) **Table S3**a, b Ceramide d18:1 (Cer) **Table S4**a-c Lysophosphatidylcholine (LPC) **Table S5**a–e Phosphatidylcholine (PC). **Table S6**a–d Phosphatidylethanolamine (PE). **Table S7**a-c Phosphatidylserine (PS) **Table S8**a-c Phosphatidylinositol (PI). (DOCX 104 kb)

## Data Availability

The datasets used or analyzed during the current study are available as supplementary table.

## Abbreviations

CE: Cholesteryl ester
Cer: Ceramide
FC: Free cholesterol
FELASA: Federation of European Laboratory Animal Science Associations
IL-6: Interleukin-6
LPC: Lysophosphatidylcholine
MCD: Methionine-choline-deficient
MDA: Malondialdehyde
MUFA: Monounsaturated
NAFLD: Non-alcoholic fatty liver disease
NASH: Non-alcoholic steatohepatitis
PC: Phosphatidylcholine
PE: Phosphatidylethanolamine
PI: Phosphatidylinositol
PS: Phosphatidylserine
PUFA: Polyunsaturated
SM: Sphingomyelin
SMA: Smooth muscle actin
TNF: Tumor necrosis factor
WT: Wild type
