# Supplementary material for: Variations in hepatic lipid species of age-matched male mice fed a methionine-choline-deficient diet and housed in different animal facilities
Source: Lipids Health Dis. 2019 Sep 14;18:172. doi: 10.1186/s12944-019-1114-4 (PMC6745065; doi:10.1186/s12944-019-1114-4)
Supplement: Supplementary file 1 — Tables. Lipids in the liver of WT1 and WT2 mice fed a control diet (WT1, WT2) or MCD diet (WT1 MCD, WT2 MCD). Concentrations are given as nmol/mg wet weight. The p-values C-MCD WT1 and C-MCD WT2 apply to the comparisons of normal to NASH liver in either WT1 or WT2 groups. C is the comparison of lipid species in the livers of WT1 and WT2 mice fed the control chow and MCD is the comparison of lipid species in the livers of WT1 and WT2 mice fed the MCD diet. * p < 0.05, ** p < 0.01. Monounsaturated, MUFA; polyunsaturated, PUFA; Sat; Saturated, Table S1a-c. Cholesteryl ester (CE) in the liver Table S2a, b Sphingomyelin (SM) Table S3a, b Ceramide d18:1 (Cer) Table S4a-c Lysophosphatidylcholine (LPC) Table S5a–e Phosphatidylcholine (PC). Table S6a–d Phosphatidylethanolamine (PE). Table S7a-c Phosphatidylserine (PS) Table S8a-c Phosphatidylinositol (PI). (DOCX 104 kb) [file 12944_2019_1114_MOESM1_ESM.docx]

**Additional files**

Table S1a: Cholesteryl ester (CE) in the liver of WT1 and WT2 mice fed a control (C, WT1, WT2) or MCD diet (WT1 MCD, WT2 MCD). Concentrations are given as nmol/mg wet weight. The p-values C-MCD WT1 and C-MCD WT2 apply to the comparisons of normal to NASH liver in either WT1 or WT2 group. C is the comparison of lipid species in the livers of WT1 and WT2 mice fed the control chow and MCD is the comparison of lipid species in the livers of WT1 and WT2 mice fed the MCD diet.* p < 0.05, ** p < 0.01.

|  | Species | CE 14:0 | CE 16:1 | CE 16:0 | CE 18:3 | CE 18:2 | CE 18:1 |
| --- | --- | --- | --- | --- | --- | --- | --- |
| WT1 | Median | 0.003 | 0.101 | 0.089 | 0.010 | 0.259 | 0.404 |
|  | Minimum | 0.002 | 0.055 | 0.063 | 0.009 | 0.226 | 0.255 |
|  | Maximum | 0.006 | 0.225 | 0.146 | 0.012 | 0.292 | 0.869 |
| WT1 MCD | Median | 0.006 | 0.089 | 0.136 | 0.013 | 0.369 | 0.471 |
|  | Minimum | 0.003 | 0.028 | 0.065 | 0.005 | 0.162 | 0.175 |
|  | Maximum | 0.015 | 0.349 | 0.585 | 0.029 | 1.358 | 2.602 |
| WT2 | Median | 0.008 | 0.170 | 0.153 | 0.019 | 0.366 | 0.503 |
|  | Minimum | 0.006 | 0.096 | 0.108 | 0.018 | 0.326 | 0.371 |
|  | Maximum | 0.009 | 0.195 | 0.180 | 0.022 | 0.387 | 0.546 |
| WT2 MCD | Median | 0.012 | 0.208 | 0.328 | 0.025 | 0.787 | 1.203 |
|  | Minimum | 0.011 | 0.165 | 0.263 | 0.022 | 0.619 | 1.009 |
|  | Maximum | 0.018 | 0.291 | 0.415 | 0.034 | 1.047 | 1.762 |
| P-value | C-MCD WT1 |  |  |  |  |  |  |
|  | C-MCD WT2 | * |  | * | * | * | * |
|  | C |  |  |  | ** | ** |  |
|  | MCD | * | * | * | * | * | * |

Table S1b: Cholesteryl ester (CE) in the liver of WT1 and WT2 mice fed a control (C, WT1, WT2) or MCD diet (WT1 MCD, WT2 MCD). Concentrations are given as nmol/mg wet weight. The p-values C-MCD WT1 and C-MCD WT2 apply to the comparisons of normal to NASH liver in either WT1 or WT2 group. C is the comparison of lipid species in the livers of WT1 and WT2 mice fed the control chow and MCD is the comparison of lipid species in the livers of WT1 and WT2 mice fed the MCD diet.* p < 0.05, ** p < 0.01. Saturated, Sat; monounsaturated, MUFA.

|  | Species | CE 18: 0 | CE 20:4 | CE 20:0 | CE 22:6 | CE Sat | CE MUFA |
| --- | --- | --- | --- | --- | --- | --- | --- |
| WT1 | Median | 0.024 | 0.085 | 0.013 | 0.016 | 0.129 | 0.509 |
|  | Minimum | 0.015 | 0.071 | 0.011 | 0.012 | 0.093 | 0.313 |
|  | Maximum | 0.039 | 0.120 | 0.015 | 0.018 | 0.206 | 1.101 |
| WT1 MCD | Median | 0.047 | 0.071 | 0.012 | 0.033 | 0.209 | 0.575 |
|  | Minimum | 0.026 | 0.035 | 0.008 | 0.023 | 0.107 | 0.206 |
|  | Maximum | 0.210 | 0.108 | 0.016 | 0.054 | 0.829 | 2.968 |
| WT2 | Median | 0.029 | 0.121 | 0.012 | 0.032 | 0.203 | 0.703 |
|  | Minimum | 0.024 | 0.111 | 0.010 | 0.028 | 0.160 | 0.468 |
|  | Maximum | 0.030 | 0.144 | 0.017 | 0.038 | 0.232 | 0.719 |
| WT2 MCD | Median | 0.088 | 0.086 | 0.015 | 0.036 | 0.439 | 1.418 |
|  | Minimum | 0.068 | 0.075 | 0.012 | 0.028 | 0.361 | 1.180 |
|  | Maximum | 0.110 | 0.103 | 0.017 | 0.040 | 0.550 | 2.054 |
| P-value | C-MCD WT1 |  |  |  | ** |  |  |
|  | C-MCD WT2 | * |  |  |  | * | * |
|  | C |  |  |  | ** |  |  |
|  | MCD |  |  |  |  | * | * |

Table S1c Cholesteryl ester (CE) and free cholesterol (FC) in the liver of WT1 and WT2 mice fed a control (C, WT1, WT2) or MCD diet (WT1 MCD, WT2 MCD). Concentrations are given as nmol/mg wet weight. The p-values C-MCD WT1 and C-MCD WT2 apply to the comparisons of normal to NASH liver in either WT1 or WT2 group. C is the comparison of lipid species in the livers of WT1 and WT2 mice fed the control chow and MCD is the comparison of lipid species in the livers of WT1 and WT2 mice fed the MCD diet. * p < 0.05, ** p < 0.01. Polyunsaturated, PUFA.

|  | Species | CE PUFA | FC |
| --- | --- | --- | --- |
| WT1 | Median | 0.380 | 5.529 |
|  | Minimum | 0.332 | 5.046 |
|  | Maximum | 0.446 | 6.759 |
| WT1 MCD | Median | 0.502 | 5.037 |
|  | Minimum | 0.234 | 3.796 |
|  | Maximum | 1.629 | 5.731 |
| WT2 | Median | 0.554 | 5.534 |
|  | Minimum | 0.506 | 5.416 |
|  | Maximum | 0.578 | 6.035 |
| WT2 MCD | Median | 0.961 | 5.909 |
|  | Minimum | 0.767 | 5.476 |
|  | Maximum | 1.246 | 8.209 |
| P-value | C-MCD WT1 |  |  |
|  | C-MCD WT2 | * |  |
|  | C | ** |  |
|  | MCD | * | * |

Table S2a Sphingomyelin (SM) in the liver of WT1 and WT2 mice fed a control (C, WT1, WT2) or MCD diet (WT1 MCD, WT2 MCD). Concentrations are given as nmol/mg wet weight. The p-values C-MCD WT1 and C-MCD WT2 apply to the comparisons of normal to NASH liver in either WT1 or WT2 group. C is the comparison of lipid species in the livers of WT1 and WT2 mice fed the control chow and MCD is the comparison of lipid species in the livers of WT1 and WT2 mice fed the MCD diet.* p < 0.05, ** p < 0.01.

|  | Species | SM 34:1 | SM 36:1 | SM 40:2 | SM 40:1 | SM 42:2 | SM 42:1 |
| --- | --- | --- | --- | --- | --- | --- | --- |
| WT1 | Median | 0.076 | 0.014 | 0.018 | 0.198 | 0.086 | 0.101 |
|  | Minimum | 0.061 | 0.010 | 0.012 | 0.164 | 0.068 | 0.087 |
|  | Maximum | 0.085 | 0.016 | 0.027 | 0.242 | 0.108 | 0.149 |
| WT1 MCD | Median | 0.087 | 0.023 | 0.006 | 0.102 | 0.075 | 0.085 |
|  | Minimum | 0.053 | 0.016 | 0.005 | 0.061 | 0.045 | 0.065 |
|  | Maximum | 0.104 | 0.032 | 0.008 | 0.117 | 0.097 | 0.105 |
| WT2 | Median | 0.083 | 0.020 | 0.013 | 0.177 | 0.080 | 0.096 |
|  | Minimum | 0.074 | 0.017 | 0.012 | 0.176 | 0.077 | 0.088 |
|  | Maximum | 0.096 | 0.023 | 0.017 | 0.214 | 0.102 | 0.103 |
| WT2 MCD | Median | 0.124 | 0.044 | 0.005 | 0.077 | 0.092 | 0.087 |
|  | Minimum | 0.112 | 0.036 | 0.001 | 0.066 | 0.076 | 0.076 |
|  | Maximum | 0.161 | 0.049 | 0.008 | 0.089 | 0.126 | 0.105 |
| P-value | C-MCD WT1 |  | ** | ** | ** |  |  |
|  | C-MCD WT2 | * | * | * | * |  |  |
|  | C |  | ** |  |  |  |  |
|  | MCD | ** | ** |  |  | * |  |

Table S2b Sphingomyelin (SM) in the liver of WT1 and WT2 mice fed a control (C, WT1, WT2) or MCD diet (WT1 MCD, WT2 MCD). Concentrations are given as nmol/mg wet weight. The p-values C-MCD WT1 and C-MCD WT2 apply to the comparisons of normal to NASH liver in either WT1 or WT2 group. C is the comparison of lipid species in the livers of WT1 and WT2 mice fed the control chow and MCD is the comparison of lipid species in the livers of WT1 and WT2 mice fed the MCD diet. ** p < 0.01. Saturated, Sat; monounsaturated, MUFA.

|  | Species | SM Sat | SM MUFA |
| --- | --- | --- | --- |
| WT1 | Median | 0.402 | 0.110 |
|  | Minimum | 0.346 | 0.084 |
|  | Maximum | 0.496 | 0.140 |
| WT1 MCD | Median | 0.297 | 0.087 |
|  | Minimum | 0.235 | 0.052 |
|  | Maximum | 0.363 | 0.107 |
| WT2 | Median | 0.388 | 0.098 |
|  | Minimum | 0.364 | 0.094 |
|  | Maximum | 0.425 | 0.125 |
| WT2 MCD | Median | 0.341 | 0.104 |
|  | Minimum | 0.312 | 0.082 |
|  | Maximum | 0.418 | 0.141 |
| P-value | C-MCD WT1 | ** |  |
|  | C-MCD WT2 |  |  |
|  | C |  |  |
|  | MCD |  |  |

Table S3a Ceramide d18:1 (Cer) in the liver of WT1 and WT2 mice fed a control (C, WT1, WT2) or MCD diet (WT1 MCD, WT2 MCD). Concentrations are given as nmol/mg wet weight. The p-values C-MCD WT1 and C-MCD WT2 apply to the comparisons of normal to NASH liver in either WT1 or WT2 group. C is the comparison of lipid species in the livers of WT1 and WT2 mice fed the control chow and MCD is the comparison of lipid species in the livers of WT1 and WT2 mice fed the MCD diet.* p < 0.05, ** p < 0.01.

|  | Species | Cer 16:0 | Cer 18:0 | Cer 20:0 | Cer 22:0 | Cer 23:0 | Cer 24:1 |
| --- | --- | --- | --- | --- | --- | --- | --- |
| WT1 | Median | 0.033 | 0.005 | 0.015 | 0.127 | 0.022 | 0.102 |
|  | Minimum | 0.026 | 0.004 | 0.012 | 0.097 | 0.018 | 0.095 |
|  | Maximum | 0.055 | 0.008 | 0.021 | 0.190 | 0.038 | 0.173 |
| WT1 MCD | Median | 0.083 | 0.021 | 0.028 | 0.128 | 0.047 | 0.210 |
|  | Minimum | 0.050 | 0.015 | 0.015 | 0.068 | 0.029 | 0.136 |
|  | Maximum | 0.109 | 0.027 | 0.036 | 0.169 | 0.060 | 0.253 |
| WT2 | Median | 0.035 | 0.009 | 0.015 | 0.115 | 0.024 | 0.105 |
|  | Minimum | 0.030 | 0.007 | 0.013 | 0.110 | 0.021 | 0.099 |
|  | Maximum | 0.067 | 0.013 | 0.025 | 0.173 | 0.027 | 0.162 |
| WT2 MCD | Median | 0.085 | 0.032 | 0.023 | 0.075 | 0.044 | 0.183 |
|  | Minimum | 0.066 | 0.020 | 0.018 | 0.059 | 0.038 | 0.152 |
|  | Maximum | 0.125 | 0.038 | 0.029 | 0.100 | 0.062 | 0.274 |
| P-value | C-MCD WT1 | ** | ** |  |  |  |  |
|  | C-MCD WT2 | * | * |  | * | * | * |
|  | C |  |  |  |  |  |  |
|  | MCD |  | * |  |  |  |  |

Table S3b Ceramide d18:1 (Cer) in the liver of WT1 and WT2 mice fed a control (C, WT1, WT2) or MCD diet (WT1 MCD, WT2 MCD). Concentrations are given as nmol/mg wet weight. The p-values C-MCD WT1 and C-MCD WT2 apply to the comparisons of normal to NASH liver in either WT1 or WT2 group. C is the comparison of lipid species in the livers of WT1 and WT2 mice fed the control chow and MCD is the comparison of lipid species in the livers of WT1 and WT2 mice fed the MCD diet. Saturated, Sat.

|  | Species | Cer 24:0 | Cer Sat |
| --- | --- | --- | --- |
| WT1 | Median | 0.076 | 0.275 |
|  | Minimum | 0.058 | 0.216 |
|  | Maximum | 0.126 | 0.438 |
| WT1 MCD | Median | 0.137 | 0.452 |
|  | Minimum | 0.076 | 0.275 |
|  | Maximum | 0.148 | 0.549 |
| WT2 | Median | 0.071 | 0.267 |
|  | Minimum | 0.068 | 0.255 |
|  | Maximum | 0.101 | 0.407 |
| WT2 MCD | Median | 0.097 | 0.358 |
|  | Minimum | 0.081 | 0.282 |
|  | Maximum | 0.124 | 0.478 |
| P-value | C-MCD WT1 |  |  |
|  | C-MCD WT2 |  |  |
|  | C |  |  |
|  | MCD |  |  |

Table S4a Lysophosphatidylcholine (LPC) in the liver of WT1 and WT2 mice fed a control (C, WT1, WT2) or MCD diet (WT1 MCD, WT2 MCD). Concentrations are given as nmol/mg wet weight. The p-values C-MCD WT1 and C-MCD WT2 apply to the comparisons of normal to NASH liver in either WT1 or WT2 group. C is the comparison of lipid species in the livers of WT1 and WT2 mice fed the control chow and MCD is the comparison of lipid species in the livers of WT1 and WT2 mice fed the MCD diet. * p < 0.05.

|  | Species | LPC 16:1 | LPC 16:0 | LPC 18:3 | LPC 18:2 | LPC 18:1 | LPC 18:0 |
| --- | --- | --- | --- | --- | --- | --- | --- |
| WT1 | Median | 0.018 | 0.101 | 0.045 | 1.406 | 0.178 | 0.066 |
|  | Minimum | 0.007 | 0.086 | 0.012 | 0.320 | 0.056 | 0.059 |
|  | Maximum | 0.045 | 0.117 | 0.062 | 1.749 | 0.474 | 0.087 |
| WT1 MCD | Median | 0.007 | 0.082 | 0.016 | 0.488 | 0.068 | 0.063 |
|  | Minimum | 0.002 | 0.051 | 0.006 | 0.118 | 0.020 | 0.043 |
|  | Maximum | 0.015 | 0.108 | 0.069 | 1.509 | 0.157 | 0.069 |
| WT2 | Median | 0.016 | 0.120 | 0.025 | 0.594 | 0.096 | 0.047 |
|  | Minimum | 0.009 | 0.098 | 0.007 | 0.198 | 0.054 | 0.041 |
|  | Maximum | 0.027 | 0.177 | 0.071 | 2.046 | 0.258 | 0.068 |
| WT2 MCD | Median | 0.014 | 0.134 | 0.046 | 1.138 | 0.173 | 0.060 |
|  | Minimum | 0.012 | 0.096 | 0.025 | 0.550 | 0.103 | 0.041 |
|  | Maximum | 0.025 | 0.170 | 0.106 | 2.503 | 0.351 | 0.078 |
| P-value | C-MCD WT1 |  |  |  |  |  |  |
|  | C-MCD WT2 |  |  |  |  |  |  |
|  | C |  |  |  |  |  |  |
|  | MCD |  | * |  |  | * |  |

Table S4b Lysophosphatidylcholine (LPC) in the liver of WT1 and WT2 mice fed a control (C, WT1, WT2) or MCD diet (WT1 MCD, WT2 MCD). Concentrations are given as nmol/mg wet weight. The p-values C-MCD WT1 and C-MCD WT2 apply to the comparisons of normal to NASH liver in either WT1 or WT2 group. C is the comparison of lipid species in the livers of WT1 and WT2 mice fed the control chow and MCD is the comparison of lipid species in the livers of WT1 and WT2 mice fed the MCD diet. * p < 0.05. Saturated, Sat.

|  | Species | LPC 20:4 | LPC 20:3 | LPC 22:6 | LPC 22:5 | LPC 22:4 | LPC Sat |
| --- | --- | --- | --- | --- | --- | --- | --- |
| WT1 | Median | 0.833 | 0.112 | 0.331 | 0.052 | 0.013 | 0.170 |
|  | Minimum | 0.214 | 0.021 | 0.094 | 0.014 | 0.004 | 0.149 |
|  | Maximum | 1.836 | 0.295 | 0.644 | 0.142 | 0.023 | 0.208 |
| WT1 MCD | Median | 0.489 | 0.029 | 0.221 | 0.035 | 0.011 | 0.148 |
|  | Minimum | 0.074 | 0.006 | 0.036 | 0.005 | 0.003 | 0.104 |
|  | Maximum | 1.440 | 0.082 | 0.826 | 0.055 | 0.018 | 0.180 |
| WT2 | Median | 0.276 | 0.020 | 0.227 | 0.024 | 0.006 | 0.180 |
|  | Minimum | 0.071 | 0.005 | 0.045 | 0.004 | 0.001 | 0.146 |
|  | Maximum | 0.764 | 0.051 | 0.734 | 0.064 | 0.015 | 0.262 |
| WT2 MCD | Median | 0.368 | 0.017 | 0.382 | 0.059 | 0.014 | 0.203 |
|  | Minimum | 0.253 | 0.009 | 0.170 | 0.023 | 0.007 | 0.144 |
|  | Maximum | 0.885 | 0.033 | 0.825 | 0.143 | 0.029 | 0.254 |
| P-value | C-MCD WT1 |  |  |  |  |  |  |
|  | C-MCD WT2 |  |  |  |  |  |  |
|  | C |  |  |  |  |  |  |
|  | MCD |  |  |  |  |  | * |

Table S4c Lysophosphatidylcholine (LPC) in the liver of WT1 and WT2 mice fed a control (C, WT1, WT2) or MCD diet (WT1 MCD, WT2 MCD). Concentrations are given as nmol/mg wet weight. The p-values C-MCD WT1 and C-MCD WT2 apply to the comparisons of normal to NASH liver in either WT1 or WT2 group. C is the comparison of lipid species in the livers of WT1 and WT2 mice fed the control chow and MCD is the comparison of lipid species in the livers of WT1 and WT2 mice fed the MCD diet. * p < 0.05. Monounsaturated, MUFA; polyunsaturated, PUFA.

|  | Species | LPC MUFA | LPC PUFA |
| --- | --- | --- | --- |
| WT1 | Median | 0.196 | 2.892 |
|  | Minimum | 0.063 | 0.713 |
|  | Maximum | 0.519 | 4.744 |
| WT1 MCD | Median | 0.074 | 1.322 |
|  | Minimum | 0.023 | 0.258 |
|  | Maximum | 0.173 | 4.001 |
| WT2 | Median | 0.112 | 1.157 |
|  | Minimum | 0.063 | 0.334 |
|  | Maximum | 0.285 | 3.751 |
| WT2 MCD | Median | 0.188 | 2.027 |
|  | Minimum | 0.115 | 1.038 |
|  | Maximum | 0.376 | 4.529 |
| P-value | C-MCD WT1 |  |  |
|  | C-MCD WT2 |  |  |
|  | C |  |  |
|  | MCD | * |  |

Table S5a Phosphatidylcholine (PC) in the liver of WT1 and WT2 mice fed a control (C, WT1, WT2) or MCD diet (WT1 MCD, WT2 MCD). Concentrations are given as nmol/mg wet weight. The p-values C-MCD WT1 and C-MCD WT2 apply to the comparisons of normal to NASH liver in either WT1 or WT2 group. C is the comparison of lipid species in the livers of WT1 and WT2 mice fed the control chow and MCD is the comparison of lipid species in the livers of WT1 and WT2 mice fed the MCD diet. * p < 0.05, ** p < 0.01.

|  | Species | PC 30:0 | PC 32:2 | PC 32:1 | PC 32:0 | PC 34:3 | PC 34:2 |
| --- | --- | --- | --- | --- | --- | --- | --- |
| WT1 | Median | 0.009 | 0.019 | 0.078 | 0.208 | 0.208 | 2.332 |
|  | Minimum | 0.008 | 0.012 | 0.047 | 0.183 | 0.184 | 1.808 |
|  | Maximum | 0.010 | 0.024 | 0.095 | 0.248 | 0.331 | 3.953 |
| WT1 MCD | Median | 0.010 | 0.012 | 0.032 | 0.218 | 0.118 | 1.220 |
|  | Minimum | 0.009 | 0.005 | 0.025 | 0.173 | 0.033 | 0.651 |
|  | Maximum | 0.012 | 0.038 | 0.056 | 0.267 | 0.376 | 3.323 |
| WT2 | Median | 0.010 | 0.023 | 0.080 | 0.220 | 0.260 | 3.047 |
|  | Minimum | 0.008 | 0.014 | 0.061 | 0.191 | 0.217 | 2.372 |
|  | Maximum | 0.011 | 0.025 | 0.096 | 0.253 | 0.278 | 3.356 |
| WT2 MCD | Median | 0.014 | 0.039 | 0.057 | 0.214 | 0.252 | 2.739 |
|  | Minimum | 0.011 | 0.024 | 0.051 | 0.201 | 0.226 | 1.786 |
|  | Maximum | 0.017 | 0.051 | 0.080 | 0.322 | 0.363 | 3.486 |
| P-value | C-MCD WT1 |  |  | ** |  |  |  |
|  | C-MCD WT2 | * | * | * |  |  |  |
|  | C |  |  |  |  |  |  |
|  | MCD | * | * | * |  |  |  |

Table S5b Phosphatidylcholine (PC) in the liver of WT1 and WT2 mice fed a control (C, WT1, WT2) or MCD diet (WT1 MCD, WT2 MCD). Concentrations are given as nmol/mg wet weight. The p-values C-MCD WT1 and C-MCD WT2 apply to the comparisons of normal to NASH liver in either WT1 or WT2 group. C is the comparison of lipid species in the livers of WT1 and WT2 mice fed the control chow and MCD is the comparison of lipid species in the livers of WT1 and WT2 mice fed the MCD diet. * p < 0.05, ** p < 0.01.

|  | Species | PC 34:1 | PC 34:0 | PC 36:5 | PC 36:4 | PC 36:3 | PC 36:2 |
| --- | --- | --- | --- | --- | --- | --- | --- |
| WT1 | Median | 0.829 | 0.020 | 0.095 | 2.003 | 0.475 | 0.925 |
|  | Minimum | 0.543 | 0.018 | 0.066 | 1.511 | 0.340 | 0.839 |
|  | Maximum | 1.043 | 0.024 | 0.115 | 2.569 | 0.569 | 1.474 |
| WT1 MCD | Median | 0.361 | 0.023 | 0.059 | 1.231 | 0.257 | 0.727 |
|  | Minimum | 0.267 | 0.021 | 0.016 | 0.827 | 0.166 | 0.463 |
|  | Maximum | 0.602 | 0.028 | 0.079 | 1.867 | 0.692 | 1.475 |
| WT2 | Median | 0.845 | 0.025 | 0.058 | 1.433 | 0.366 | 0.746 |
|  | Minimum | 0.707 | 0.021 | 0.046 | 1.138 | 0.312 | 0.565 |
|  | Maximum | 0.905 | 0.028 | 0.072 | 1.834 | 0.470 | 0.858 |
| WT2 MCD | Median | 0.766 | 0.021 | 0.050 | 1.342 | 0.731 | 1.158 |
|  | Minimum | 0.725 | 0.017 | 0.047 | 1.012 | 0.630 | 0.829 |
|  | Maximum | 1.106 | 0.028 | 0.077 | 1.999 | 1.034 | 1.476 |
| p-values | C-MCD WT1 | ** |  |  |  |  |  |
|  | C-MCD WT2 |  |  |  |  | * | * |
|  | C |  |  | * | * |  | * |
|  | MCD | ** |  |  |  | * | * |

Table S5c Phosphatidylcholine (PC) in the liver of WT1 and WT2 mice fed a control (C, WT1, WT2) or MCD diet (WT1 MCD, WT2 MCD). Concentrations are given as nmol/mg wet weight. The p-values C-MCD WT1 and C-MCD WT2 apply to the comparisons of normal to NASH liver in either WT1 or WT2 group. C is the comparison of lipid species in the livers of WT1 and WT2 mice fed the control chow and MCD is the comparison of lipid species in the livers of WT1 and WT2 mice fed the MCD diet. * p < 0.05, ** p < 0.01.

|  | Species | PC 36:1 | PC 38:6 | PC 38:5 | PC 38:4 | PC 38:3 | PC 38:2 |
| --- | --- | --- | --- | --- | --- | --- | --- |
| WT1 | Median | 0.110 | 0.966 | 0.515 | 1.420 | 0.123 | 0.030 |
|  | Minimum | 0.077 | 0.640 | 0.325 | 0.980 | 0.083 | 0.027 |
|  | Maximum | 0.156 | 1.188 | 0.729 | 1.670 | 0.182 | 0.045 |
| WT1 MCD | Median | 0.056 | 0.795 | 0.282 | 1.279 | 0.056 | 0.014 |
|  | Minimum | 0.047 | 0.344 | 0.120 | 0.776 | 0.043 | 0.012 |
|  | Maximum | 0.089 | 1.448 | 0.380 | 1.454 | 0.137 | 0.020 |
| WT2 | Median | 0.078 | 1.115 | 0.271 | 0.715 | 0.045 | 0.013 |
|  | Minimum | 0.067 | 0.656 | 0.188 | 0.608 | 0.031 | 0.011 |
|  | Maximum | 0.155 | 1.249 | 0.334 | 1.006 | 0.056 | 0.062 |
| WT2 MCD | Median | 0.109 | 1.022 | 0.368 | 0.960 | 0.040 | 0.015 |
|  | Minimum | 0.092 | 0.908 | 0.325 | 0.672 | 0.030 | 0.008 |
|  | Maximum | 0.139 | 1.534 | 0.514 | 1.431 | 0.056 | 0.016 |
| P-value | C-MCD WT1 | ** |  |  |  | ** | ** |
|  | C-MCD WT2 |  |  |  |  |  |  |
|  | C |  |  |  |  | * |  |
|  | MCD | ** |  |  |  |  |  |

Table S5d Phosphatidylcholine (PC) in the liver of WT1 and WT2 mice fed a control (C, WT1, WT2) or MCD diet (WT1 MCD, WT2 MCD). Concentrations are given as nmol/mg wet weight. The p-values C-MCD WT1 and C-MCD WT2 apply to the comparisons of normal to NASH liver in either WT1 or WT2 group. C is the comparison of lipid species in the livers of WT1 and WT2 mice fed the control chow and MCD is the comparison of lipid species in the livers of WT1 and WT2 mice fed the MCD diet. * p < 0.05, ** p < 0.01. Saturated, Sat; Monounsaturated, MUFA.

|  | Species | PC 38:1 | PC 40:6 | PC 40:5 | PC 40:4 | PC Sat | PC MUFA |
| --- | --- | --- | --- | --- | --- | --- | --- |
| WT1 | Median | 0.007 | 0.238 | 0.070 | 0.028 | 0.242 | 1.027 |
|  | Minimum | 0.006 | 0.159 | 0.042 | 0.021 | 0.213 | 0.673 |
|  | Maximum | 0.007 | 0.299 | 0.086 | 0.037 | 0.287 | 1.301 |
| WT1 MCD | Median | 0.004 | 0.298 | 0.042 | 0.016 | 0.258 | 0.454 |
|  | Minimum | 0.001 | 0.158 | 0.024 | 0.010 | 0.212 | 0.345 |
|  | Maximum | 0.007 | 0.470 | 0.059 | 0.016 | 0.306 | 0.753 |
| WT2 | Median | 0.005 | 0.267 | 0.034 | 0.014 | 0.287 | 1.058 |
|  | Minimum | 0.003 | 0.165 | 0.027 | 0.011 | 0.225 | 0.840 |
|  | Maximum | 0.037 | 0.334 | 0.045 | 0.031 | 0.377 | 1.126 |
| WT2 MCD | Median | 0.005 | 0.344 | 0.071 | 0.014 | 0.250 | 0.934 |
|  | Minimum | 0.003 | 0.319 | 0.059 | 0.011 | 0.238 | 0.878 |
|  | Maximum | 0.006 | 0.478 | 0.081 | 0.018 | 0.377 | 1.331 |
| p-value | C-MCD WT1 | ** |  |  | ** |  | ** |
|  | C-MCD WT2 |  |  | * |  |  |  |
|  | C |  |  | * |  |  |  |
|  | MCD |  |  | ** |  |  | ** |

Table S5e Phosphatidylcholine (PC) in the liver of WT1 and WT2 mice fed a control (C, WT1, WT2) or MCD diet (WT1 MCD, WT2 MCD). Concentrations are given as nmol/mg wet weight. The p-values C-MCD WT1 and C-MCD WT2 apply to the comparisons of normal to NASH liver in either WT1 or WT2 group. C is the comparison of lipid species in the livers of WT1 and WT2 mice fed the control chow and MCD is the comparison of lipid species in the livers of WT1 and WT2 mice fed the MCD diet. Polyunsaturated, PUFA.

|  | Species | PC PUFA |
| --- | --- | --- |
| WT1 | Median | 9.516 |
|  | Minimum | 7.229 |
|  | Maximum | 13.004 |
| WT1 MCD | Median | 6.082 |
|  | Minimum | 3.736 |
|  | Maximum | 11.732 |
| WT2 | Median | 8.691 |
|  | Minimum | 6.825 |
|  | Maximum | 9.487 |
| WT2 MCD | Median | 9.214 |
|  | Minimum | 6.898 |
|  | Maximum | 12.062 |
| p-value | C-MCD WT1 |  |
|  | C-MCD WT2 |  |
|  | C |  |
|  | MCD |  |

Table S6a Phosphatidylethanolamine (PE) in the liver of WT1 and WT2 mice fed a control (C, WT1, WT2) or MCD diet (WT1 MCD, WT2 MCD). Concentrations are given as nmol/mg wet weight. The p-values C-MCD WT1 and C-MCD WT2 apply to the comparisons of normal to NASH liver in either WT1 or WT2 group. C is the comparison of lipid species in the livers of WT1 and WT2 mice fed the control chow and MCD is the comparison of lipid species in the livers of WT1 and WT2 mice fed the MCD diet. * p < 0.05, ** p< 0.01.

|  | Species | PE 34:3 | PE 34:2 | PE 34:1 | PE 36:5 | PE 36:4 | PE 36:3 |
| --- | --- | --- | --- | --- | --- | --- | --- |
| WT1 | Median | 0.024 | 0.269 | 0.033 | 0.035 | 0.426 | 0.150 |
|  | Minimum | 0.019 | 0.181 | 0.025 | 0.028 | 0.386 | 0.114 |
|  | Maximum | 0.039 | 0.453 | 0.041 | 0.040 | 0.583 | 0.221 |
| WT1 MCD | Median | 0.017 | 0.207 | 0.025 | 0.015 | 0.302 | 0.123 |
|  | Minimum | 0.009 | 0.139 | 0.019 | 0.006 | 0.242 | 0.069 |
|  | Maximum | 0.028 | 0.281 | 0.038 | 0.019 | 0.355 | 0.231 |
| WT2 | Median | 0.031 | 0.400 | 0.035 | 0.021 | 0.400 | 0.178 |
|  | Minimum | 0.031 | 0.367 | 0.027 | 0.018 | 0.258 | 0.147 |
|  | Maximum | 0.039 | 0.457 | 0.041 | 0.026 | 0.493 | 0.222 |
| WT2 MCD | Median | 0.018 | 0.232 | 0.037 | 0.010 | 0.272 | 0.233 |
|  | Minimum | 0.016 | 0.190 | 0.026 | 0.009 | 0.254 | 0.204 |
|  | Maximum | 0.025 | 0.296 | 0.050 | 0.014 | 0.400 | 0.320 |
| P-value | C-MCD WT1 |  |  |  | ** | ** |  |
|  | C-MCD WT2 |  | * |  | * |  |  |
|  | C |  |  |  | * |  |  |
|  | MCD |  |  |  |  |  | * |

Table S6b Phosphatidylethanolamine (PE) in the liver of WT1 and WT2 mice fed a control (C, WT1, WT2) or MCD diet (WT1 MCD, WT2 MCD). Concentrations are given as nmol/mg wet weight. The p-values C-MCD WT1 and C-MCD WT2 apply to the comparisons of normal to NASH liver in either WT1 or WT2 group. C is the comparison of lipid species in the livers of WT1 and WT2 mice fed the control chow and MCD is the comparison of lipid species in the livers of WT1 and WT2 mice fed the MCD diet. * p < 0.05, ** p< 0.01.

|  | Species | PE 36:2 | PE 36:1 | PE 38:6 | PE 38:5 | PE 38:4 | PE 38:3 |
| --- | --- | --- | --- | --- | --- | --- | --- |
| WT1 | Median | 0.221 | 0.010 | 0.892 | 0.611 | 1.843 | 0.039 |
|  | Minimum | 0.154 | 0.009 | 0.814 | 0.488 | 1.695 | 0.032 |
|  | Maximum | 0.354 | 0.014 | 1.158 | 0.705 | 2.348 | 0.050 |
| WT1 MCD | Median | 0.192 | 0.011 | 1.123 | 0.312 | 1.229 | 0.015 |
|  | Minimum | 0.135 | 0.009 | 0.848 | 0.224 | 0.803 | 0.010 |
|  | Maximum | 0.348 | 0.017 | 1.290 | 0.351 | 1.640 | 0.040 |
| WT2 | Median | 0.262 | 0.010 | 1.078 | 0.331 | 1.625 | 0.017 |
|  | Minimum | 0.243 | 0.006 | 0.592 | 0.216 | 1.132 | 0.014 |
|  | Maximum | 0.333 | 0.012 | 1.345 | 0.423 | 1.920 | 0.026 |
| WT2 MCD | Median | 0.352 | 0.023 | 0.977 | 0.353 | 1.266 | 0.019 |
|  | Minimum | 0.303 | 0.014 | 0.920 | 0.290 | 1.128 | 0.014 |
|  | Maximum | 0.447 | 0.030 | 1.425 | 0.486 | 1.679 | 0.022 |
| P-value | C-MCD WT1 |  |  |  | ** | ** | ** |
|  | C-MCD WT2 |  | * |  |  |  |  |
|  | C |  |  |  | * |  | * |
|  | MCD | * | * |  |  |  |  |

Table S6c Phosphatidylethanolamine (PE) in the liver of WT1 and WT2 mice fed a control (C, WT1, WT2) or MCD diet (WT1 MCD, WT2 MCD). Concentrations are given as nmol/mg wet weight. The p-values C-MCD WT1 and C-MCD WT2 apply to the comparisons of normal to NASH liver in either WT1 or WT2 group. C is the comparison of lipid species in the livers of WT1 and WT2 mice fed the control chow and MCD is the comparison of lipid species in the livers of WT1 and WT2 mice fed the MCD diet. * p < 0.05, ** p < 0.01.

|  | Species | PE 38:2 | PE 40:7 | PE 40:6 | PE 40:5 | PE 40:4 | PE 40:3 |
| --- | --- | --- | --- | --- | --- | --- | --- |
| WT1 | Median | 0.014 | 0.685 | 0.793 | 0.212 | 0.107 | 0.004 |
|  | Minimum | 0.012 | 0.509 | 0.711 | 0.147 | 0.092 | 0.004 |
|  | Maximum | 0.020 | 0.863 | 1.173 | 0.258 | 0.155 | 0.007 |
| WT1 MCD | Median | 0.006 | 0.927 | 1.700 | 0.205 | 0.066 | 0.001 |
|  | Minimum | 0.005 | 0.448 | 1.389 | 0.103 | 0.046 | 0.001 |
|  | Maximum | 0.011 | 1.055 | 1.957 | 0.285 | 0.079 | 0.002 |
| WT2 | Median | 0.006 | 0.659 | 1.283 | 0.151 | 0.083 | 0.003 |
|  | Minimum | 0.006 | 0.332 | 0.796 | 0.118 | 0.065 | 0.002 |
|  | Maximum | 0.010 | 0.799 | 1.767 | 0.215 | 0.095 | 0.010 |
| WT2 MCD | Median | 0.006 | 0.844 | 1.871 | 0.330 | 0.068 | 0.002 |
|  | Minimum | 0.005 | 0.795 | 1.684 | 0.237 | 0.057 | 0.001 |
|  | Maximum | 0.009 | 1.230 | 2.600 | 0.400 | 0.086 | 0.003 |
| P-value | C-MCD WT1 | ** |  | ** |  | ** | ** |
|  | C-MCD WT2 |  | * | * | * |  |  |
|  | C | * |  |  |  | * |  |
|  | MCD |  |  | * | * |  |  |

Table S6d Phosphatidylethanolamine (PE) in the liver of WT1 and WT2 mice fed a control (C, WT1, WT2) or MCD diet (WT1 MCD, WT2 MCD). Concentrations are given as nmol/mg wet weight. The p-values C-MCD WT1 and C-MCD WT2 apply to the comparisons of normal to NASH liver in either WT1 or WT2 group. C is the comparison of lipid species in the livers of WT1 and WT2 mice fed the control chow and MCD is the comparison of lipid species in the livers of WT1 and WT2 mice fed the MCD diet. * p < 0.05, ** p < 0.01. Monounsaturated, MUFA; polyunsaturated, PUFA.

|  | Species | PE 42:7 | PE 42:6 | PE MUFA | PE PUFA |
| --- | --- | --- | --- | --- | --- |
| WT1 | Median | 0.018 | 0.012 | 0.044 | 6.167 |
|  | Minimum | 0.017 | 0.010 | 0.035 | 5.739 |
|  | Maximum | 0.026 | 0.020 | 0.056 | 8.439 |
| WT1 MCD | Median | 0.015 | 0.007 | 0.035 | 6.612 |
|  | Minimum | 0.013 | 0.005 | 0.026 | 5.363 |
|  | Maximum | 0.019 | 0.010 | 0.053 | 7.373 |
| WT2 | Median | 0.015 | 0.007 | 0.048 | 6.446 |
|  | Minimum | 0.009 | 0.005 | 0.034 | 4.396 |
|  | Maximum | 0.017 | 0.008 | 0.055 | 8.098 |
| WT2 MCD | Median | 0.016 | 0.006 | 0.061 | 6.889 |
|  | Minimum | 0.014 | 0.004 | 0.038 | 6.522 |
|  | Maximum | 0.020 | 0.006 | 0.080 | 9.481 |
| P-value | C-MCD WT1 |  | ** |  |  |
|  | C-MCD WT2 |  |  |  |  |
|  | C |  | * |  |  |
|  | MCD |  |  |  |  |

Table S7a Phosphatidylserine (PS) in the liver of WT1 and WT2 mice mice fed a control (C, WT1, WT2) or MCD diet (WT1 MCD, WT2 MCD). Concentrations are given as nmol/mg wet weight. The p-values C-MCD WT1 and C-MCD WT2 apply to the comparisons of normal to NASH liver in either WT1 or WT2 group. C is the comparison of lipid species in the livers of WT1 and WT2 mice fed the control chow and MCD is the comparison of lipid species in the livers of WT1 and WT2 mice fed the MCD diet. * p < 0.05, ** p < 0.01.

|  | Species | PS 36:4 | PS 36:2 | PS 36:1 | PS 38:6 | PS 38:5 | PS 38:4 |
| --- | --- | --- | --- | --- | --- | --- | --- |
| WT1 | Median | 0.072 | 0.029 | 0.036 | 0.062 | 0.039 | 0.964 |
|  | Minimum | 0.065 | 0.026 | 0.028 | 0.055 | 0.034 | 0.792 |
|  | Maximum | 0.095 | 0.038 | 0.038 | 0.094 | 0.052 | 1.143 |
| WT1 MCD | Median | 0.030 | 0.033 | 0.039 | 0.082 | 0.022 | 0.552 |
|  | Minimum | 0.017 | 0.024 | 0.036 | 0.049 | 0.015 | 0.315 |
|  | Maximum | 0.037 | 0.044 | 0.043 | 0.099 | 0.026 | 0.659 |
| WT2 | Median | 0.057 | 0.025 | 0.031 | 0.071 | 0.021 | 0.703 |
|  | Minimum | 0.035 | 0.022 | 0.026 | 0.046 | 0.018 | 0.543 |
|  | Maximum | 0.067 | 0.028 | 0.035 | 0.079 | 0.026 | 0.813 |
| WT2 MCD | Median | 0.022 | 0.038 | 0.038 | 0.057 | 0.018 | 0.414 |
|  | Minimum | 0.015 | 0.033 | 0.030 | 0.045 | 0.013 | 0.346 |
|  | Maximum | 0.030 | 0.048 | 0.041 | 0.087 | 0.024 | 0.536 |
| P-value | C-MCD WT1 | ** |  |  |  | ** | ** |
|  | C-MCD WT2 | * | * |  |  |  | * |
|  | C |  | * |  |  | * | * |
|  | MCD |  |  |  |  |  |  |

Table S7b Phosphatidylserine (PS) in the liver of WT1 and WT2 mice mice fed a control (C, WT1, WT2) or MCD diet (WT1 MCD, WT2 MCD). Concentrations are given as nmol/mg wet weight. The p-values C-MCD WT1 and C-MCD WT2 apply to the comparisons of normal to NASH liver in either WT1 or WT2 group. C is the comparison of lipid species in the livers of WT1 and WT2 mice fed the control chow and MCD is the comparison of lipid species in the livers of WT1 and WT2 mice fed the MCD diet. * p < 0.05, ** p < 0.01. Monounsaturated, MUFA.

|  | Species | PS 38:3 | PS 40:7 | PS 40:6 | PS 40:5 | PS 40:4 | PS MUFA |
| --- | --- | --- | --- | --- | --- | --- | --- |
| WT1 | Median | 0.038 | 0.055 | 0.348 | 0.115 | 0.078 | 0.041 |
|  | Minimum | 0.035 | 0.050 | 0.320 | 0.070 | 0.066 | 0.035 |
|  | Maximum | 0.051 | 0.060 | 0.531 | 0.137 | 0.107 | 0.047 |
| WT1 MCD | Median | 0.026 | 0.050 | 0.663 | 0.112 | 0.085 | 0.042 |
|  | Minimum | 0.024 | 0.040 | 0.498 | 0.069 | 0.067 | 0.036 |
|  | Maximum | 0.030 | 0.055 | 0.796 | 0.135 | 0.094 | 0.052 |
| WT2 | Median | 0.027 | 0.050 | 0.493 | 0.072 | 0.069 | 0.031 |
|  | Minimum | 0.019 | 0.045 | 0.355 | 0.062 | 0.043 | 0.028 |
|  | Maximum | 0.031 | 0.052 | 0.561 | 0.087 | 0.076 | 0.039 |
| WT2 MCD | Median | 0.025 | 0.049 | 0.788 | 0.153 | 0.075 | 0.038 |
|  | Minimum | 0.015 | 0.039 | 0.622 | 0.115 | 0.069 | 0.030 |
|  | Maximum | 0.033 | 0.052 | 1.037 | 0.182 | 0.103 | 0.046 |
| P-value | C-MCD WT1 | ** |  | ** |  |  |  |
|  | C-MCD WT2 |  |  | * | * |  |  |
|  | C | * |  |  | * |  | * |
|  | MCD |  |  |  | * |  |  |

Table S7c Phosphatidylserine (PS) in the liver of WT1 and WT2 mice mice fed a control (C, WT1, WT2) or MCD diet (WT1 MCD, WT2 MCD). Concentrations are given as nmol/mg wet weight. The p-values C-MCD WT1 and C-MCD WT2 apply to the comparisons of normal to NASH liver in either WT1 or WT2 group. C is the comparison of lipid species in the livers of WT1 and WT2 mice fed the control chow and MCD is the comparison of lipid species in the livers of WT1 and WT2 mice fed the MCD diet. Polyunsaturated, PUFA.

|  | Species | PS PUFA |
| --- | --- | --- |
| WT1 | Median | 1.818 |
|  | Minimum | 1.608 |
|  | Maximum | 2.326 |
| WT1 MCD | Median | 1.670 |
|  | Minimum | 1.344 |
|  | Maximum | 1.925 |
| WT2 | Median | 1.615 |
|  | Minimum | 1.202 |
|  | Maximum | 1.792 |
| WT2 MCD | Median | 1.644 |
|  | Minimum | 1.373 |
|  | Maximum | 2.137 |
| P-value | C-MCD WT1 |  |
|  | C-MCD WT2 |  |
|  | C |  |
|  | MCD |  |

Table S8a Phosphatidylinositol (PI) in the liver of WT1 and WT2 mice mice fed a control (C, WT1, WT2) or MCD diet (WT1 MCD, WT2 MCD). Concentrations are given as nmol/mg wet weight. The p-values C-MCD WT1 and C-MCD WT2 apply to the comparisons of normal to NASH liver in either WT1 or WT2 group. C is the comparison of lipid species in the livers of WT1 and WT2 mice fed the control chow and MCD is the comparison of lipid species in the livers of WT1 and WT2 mice fed the MCD diet. * p < 0.05, ** p < 0.01.

|  | Species | PI 34:2 | PI 36:4 | PI 36:3 | PI 36:2 | PI 36:1 | PI 38:6 |
| --- | --- | --- | --- | --- | --- | --- | --- |
| WT1 | Median | 0.057 | 0.247 | 0.086 | 0.116 | 0.004 | 0.022 |
|  | Minimum | 0.048 | 0.208 | 0.050 | 0.093 | 0.002 | 0.017 |
|  | Maximum | 0.096 | 0.363 | 0.111 | 0.185 | 0.006 | 0.031 |
| WT1 MCD | Median | 0.029 | 0.136 | 0.050 | 0.101 | 0.005 | 0.018 |
|  | Minimum | 0.022 | 0.121 | 0.020 | 0.065 | 0.003 | 0.016 |
|  | Maximum | 0.042 | 0.165 | 0.059 | 0.150 | 0.010 | 0.023 |
| WT2 | Median | 0.057 | 0.209 | 0.061 | 0.125 | 0.005 | 0.020 |
|  | Minimum | 0.044 | 0.138 | 0.040 | 0.105 | 0.004 | 0.008 |
|  | Maximum | 0.062 | 0.268 | 0.064 | 0.151 | 0.006 | 0.022 |
| WT2 MCD | Median | 0.026 | 0.103 | 0.058 | 0.187 | 0.014 | 0.014 |
|  | Minimum | 0.013 | 0.094 | 0.023 | 0.050 | 0.004 | 0.011 |
|  | Maximum | 0.037 | 0.162 | 0.075 | 0.242 | 0.020 | 0.023 |
| P-value | C-MCD WT1 | ** | ** |  |  |  |  |
|  | C-MCD WT2 | * | * |  |  |  |  |
|  | C |  |  |  |  |  |  |
|  | MCD |  |  |  |  |  |  |

Table S8b Phosphatidylinositol (PI) in the liver of WT1 and WT2 mice mice fed a control (C, WT1, WT2) or MCD diet (WT1 MCD, WT2 MCD). Concentrations are given as nmol/mg wet weight. The p-values C-MCD WT1 and C-MCD WT2 apply to the comparisons of normal to NASH liver in either WT1 or WT2 group. C is the comparison of lipid species in the livers of WT1 and WT2 mice fed the control chow and MCD is the comparison of lipid species in the livers of WT1 and WT2 mice fed the MCD diet. * p < 0.05, ** p < 0.01.

|  | Species | PI 38:5 | PI 38:4 | PI 38:3 | PI 40:6 | PI 40:5 | PI 40:4 |
| --- | --- | --- | --- | --- | --- | --- | --- |
| WT1 | Median | 0.257 | 4.283 | 0.170 | 0.042 | 0.031 | 0.060 |
|  | Minimum | 0.167 | 3.545 | 0.112 | 0.032 | 0.026 | 0.046 |
|  | Maximum | 0.346 | 5.189 | 0.304 | 0.050 | 0.049 | 0.076 |
| WT1 MCD | Median | 0.126 | 4.011 | 0.029 | 0.045 | 0.019 | 0.022 |
|  | Minimum | 0.041 | 2.521 | 0.004 | 0.033 | 0.018 | 0.016 |
|  | Maximum | 0.158 | 4.764 | 0.081 | 0.050 | 0.026 | 0.032 |
| WT2 | Median | 0.143 | 4.163 | 0.032 | 0.037 | 0.021 | 0.028 |
|  | Minimum | 0.073 | 2.835 | 0.018 | 0.020 | 0.013 | 0.018 |
|  | Maximum | 0.167 | 4.760 | 0.060 | 0.045 | 0.028 | 0.032 |
| WT2 MCD | Median | 0.109 | 4.005 | 0.014 | 0.039 | 0.023 | 0.016 |
|  | Minimum | 0.087 | 3.834 | 0.007 | 0.031 | 0.020 | 0.015 |
|  | Maximum | 0.157 | 6.177 | 0.021 | 0.059 | 0.035 | 0.021 |
| P-value | C-MCD WT1 | ** |  | ** |  | ** | ** |
|  | C-MCD WT2 |  |  | * |  |  | * |
|  | C | * |  | * |  |  | * |
|  | MCD |  |  |  |  |  |  |

Table S8c Phosphatidylinositol (PI) in the liver of WT1 and WT2 mice mice fed a control (C, WT1, WT2) or MCD diet (WT1 MCD, WT2 MCD). Concentrations are given as nmol/mg wet weight. The p-values C-MCD WT1 and C-MCD WT2 apply to the comparisons of normal to NASH liver in either WT1 or WT2 group. C is the comparison of lipid species in the livers of WT1 and WT2 mice fed the control chow and MCD is the comparison of lipid species in the livers of WT1 and WT2 mice fed the MCD diet. Monounsaturated, MUFA; Polyunsaturated, PUFA.

|  | Species | PI MUFA | PI PUFA |
| --- | --- | --- | --- |
| WT1 | Median | 0.009 | 5.480 |
|  | Minimum | 0.005 | 4.503 |
|  | Maximum | 0.014 | 6.601 |
| WT1 MCD | Median | 0.010 | 4.612 |
|  | Minimum | 0.006 | 3.043 |
|  | Maximum | 0.013 | 5.387 |
| WT2 | Median | 0.011 | 4.897 |
|  | Minimum | 0.009 | 3.329 |
|  | Maximum | 0.014 | 5.633 |
| WT2 MCD | Median | 0.019 | 4.677 |
|  | Minimum | 0.007 | 4.341 |
|  | Maximum | 0.026 | 7.012 |
| P-value | C-MCD WT1 |  |  |
|  | C-MCD WT2 |  |  |
|  | SD |  |  |
|  | MCD |  |  |
